# Loss of the adhesion G-protein coupled receptor ADGRF5 in mice induces airway inflammation and the expression of CCL2 in lung endothelial cells

**DOI:** 10.1186/s12931-019-0973-6

**Published:** 2019-01-17

**Authors:** Fumimasa Kubo, Donna Maretta Ariestanti, Souta Oki, Taku Fukuzawa, Ryotaro Demizu, Tomoya Sato, Rahmaningsih Mara Sabirin, Shigehisa Hirose, Nobuhiro Nakamura

**Affiliations:** 1 Department of Life Science and Technology, Tokyo Institute of Technology, 4259-B13 Nagatsuta-cho, Midori-ku, Yokohama, 226-8501 Japan; 2 Department of Physiology, Faculty of Medicine, Public Health and Nursing, Gadjah Mada University, JI.Farmako Sekip Utara, Yogyakarta, 55281 Indonesia

**Keywords:** Adhesion GPCR, Asthma, COPD, Endothelial cell, Fibrosis, GPR116, Ig-Hepta, Mucous cell metaplasia, Type 2 cytokine

## Abstract

**Background:**

Adhesion G-protein coupled receptor F5 (ADGRF5) was recently identified as an essential regulator of pulmonary surfactant homeostasis in alveolar type II cells. We previously showed that in addition to abnormal surfactant accumulation, *Adgrf5*-deficient (*Adgrf5*^*−/−*^) mice exhibit emphysema-like signs, suggesting a possible role for ADGRF5 in immune regulation. Here, we extended the phenotypic analysis of *Adgrf5*^*−/−*^ mice to help understand its biological role in the lung, and especially in immune regulation.

**Methods:**

Histological features of lungs were evaluated by Alcian blue and Masson’s trichrome staining. Quantitative real-time PCR (qPCR) and western blot analyses were performed to analyze the differential expression of genes/proteins related to airway inflammation in lungs between wildtype and *Adgrf5*^*−/−*^ mice. Acid–base status was assessed by performing blood gas tests and urine pH measurements. Inflammatory cell counting was performed using Giemsa-stained bronchoalveolar lavage cells. Serum IgE concentrations were determined by enzyme-linked immunosorbent assay. The expression of *Ccl2*, *S100a8*, *S100a9*, and *Saa3* in primary lung endothelial cells (ECs) was determined by qPCR and/or western blotting. Finally, the effect of administrating RS504393 to 2-week-old *Adgrf5*^*−/−*^ mice on gene expression in the lungs was analyzed by qPCR.

**Results:**

*Adgrf5*^*−/−*^ mice exhibited several features of chronic airway inflammation (mucous cell metaplasia, mucus hyperproduction, subepithelial fibrosis, respiratory acidosis, high serum IgE, mast cell accumulation, and neutrophilia) in parallel with elevated expression of genes involved in mucous cell metaplasia (*Muc5ac*, *Muc5b*, *Slc26a4*, and *Clca1*), fibrosis (*Tgfb1*, *Col1a1*, *Fn1*, and *Tnc*), and type 2 immune response (*Il4*, *Il5*, *Il13*, IL-25, and IL-33) at 12 and/or 30 weeks of age. In contrast, mRNA expression of *Ccl2*, *S100a8*, and *S100a9* was upregulated in embryonic or neonatal *Adgrf5*^*−/−*^ lungs as well as in lung ECs of *Adgrf5*^*−/−*^ mice at 1 week of age. RS504393 treatment suppressed the upregulation of *S100a8*, *S100a9*, *Slc26a4*, and *Il5* in *Adgrf5*^*−/−*^ lungs.

**Conclusions:**

Targeted disruption of ADGRF5 results in the development of airway inflammation, which is likely mediated by the type 2 immune response and possibly CCL2-mediated inflammation. ADGRF5 also has a potential role in the regulation of genes encoding CCL2 in lung ECs, thereby maintaining immune homeostasis.

**Electronic supplementary material:**

The online version of this article (10.1186/s12931-019-0973-6) contains supplementary material, which is available to authorized users.

## Background

Adhesion G protein-coupled receptors (GPCRs) comprise the second largest group of seven transmembrane receptors (33 members in humans), and are defined by exceptionally long N-terminal extracellular domains. These proteins are expressed in a cell type- and tissue-specific manner and are associated with diverse cellular and physiological functions such as cell adhesion and migration, immune responses, and tumorigenesis [1]. Among adhesion GPCRs, adhesion GPCR F5 (ADGRF5, also known as GPR116 or Ig-Hepta) is unique in that it contains immunoglobulin (Ig)-like repeats and an SEA module in its long extracellular domain of approximately 1000 amino acid residues [2, 3]. ADGRF5 undergoes self-cleavage, yielding a soluble N-terminal fragment and a membrane-bound C-terminal fragment, both of which are non-covalently associated with each other [3]. Although the endogenous ligand has not been identified, recent studies reported that ADGRF5 likely couples with G_q/11_ and can be activated by a short synthetic peptide located at the N-terminus of the C-terminal fragment (called the *stachel* sequence) as a tethered agonist [4–6]. ADGRF5 is expressed predominantly in the lung and to a lesser extent in many other tissues such as the heart, kidney, and adipose tissue [1, 2, 7, 8]. In the lung, ADGRF5 expression is readily detectable in alveolar type II (AT2) epithelial cells and the vascular endothelium [8–11]. It has been established that ADGRF5 is critical for maintaining pulmonary surfactant homeostasis, as targeted disruption of mouse *Adgrf5* results in the massive accumulation of surfactant lipids and proteins in the alveoli [8–11]. It has also been shown that ADGRF5 controls the surfactant pool size by suppressing the secretion and promoting the uptake of surfactant in AT2 cells via the G_q/11_ signaling pathway [6]. Moreover, the accumulation of pulmonary surfactant is also induced by epithelial-cell-specific and AT2-cell-specific deletion of *Adgrf5*, but not endothelial-cell (EC)-specific deletion of Adgrf5 [6], suggesting that the role of ADGRF5 in surfactant homeostasis is restricted to AT2 cells. Recently, loss of ADGRF5 has been shown to increase blood-brain-barrier permeability and promote retinal angiogenesis in a model of oxygen-induced retinopathy [11]. However, the function of ADGRF5 in lung ECs is not fully understood.

*Adgrf5*-deficient (*Adgrf5*^*−/−*^) mice exhibit an accumulation of foamy alveolar macrophages in the alveoli as well as emphysema-like signs including alveolar enlargement [8–12]. The expression of mouse *Adgrf5* mRNA in the lung is upregulated at 18 days post-coitum (dpc) and peaks at 1–3 weeks of age [9, 10]. In *Adgrf5*^*−/−*^ mice, excessive pulmonary surfactant can be detected at 1 week of age, and the accumulation of alveolar macrophages occurs at 2–3 weeks of age [10, 11]. In addition, the fact that ADGRF5 is not expressed in alveolar macrophages [8, 10] suggests that the accumulation of alveolar macrophages is not a direct result of *Adgrf5* deletion, but rather a secondary effect based on the increased surfactant pool size. We previously showed that alveolar macrophages from *Adgrf5*^*−/−*^ mice produce and release reactive oxygen species, matrix metalloproteases (MMPs), and proinflammatory cytokines/chemokines, which might cause alveolar tissue destruction and inflammation [12]. The major chemokines secreted from these alveolar macrophages are C-C motif chemokine ligand 2 (CCL2, also known as monocyte chemotactic protein-1 (MCP-1)), and CCL3, which likely enhance the recruitment of monocytes and macrophages to the lung. Interestingly, an increase in CCL2 levels was detected in whole lungs of *Adgrf5*^*−/−*^ mice at 18.5 dpc [12], at which time the accumulation of neither pulmonary surfactant nor alveolar macrophages had occurred [9, 10]. We therefore hypothesized that ADGRF5 might also have a role in maintaining immune homeostasis in the lung.

The lung is continuously exposed to inhaled pathogens, allergens, and environmental pollutants, and rapidly eliminates these particles with the help of the immune system. Immune responses in the lung must be tightly regulated to prevent inflammation and tissue damage. Inappropriate or excessive immune responses cause the development of chronic airway inflammation, which is a fundamental feature of chronic obstructive pulmonary disease (COPD) and asthma. COPD is caused mostly by cigarette smoking and is generally characterized by irreversible airway obstruction, mucous cell metaplasia, peribronchiolar fibrosis, lung parenchyma destruction, and neutrophil infiltration. In contrast, asthma is caused by allergy and is characterized by reversible airway hyperresponsiveness, mucous cell metaplasia, subepithelial fibrosis, and eosinophil infiltration [13]. However, up to 40% of COPD patients display eosinophilia and approximately 50% of asthmatic patients have non-eosinophilic and neutrophilic asthma [14, 15]. Extensive studies have illustrated the molecular mechanisms underlying the pathogenesis of airway inflammation. The pathogenesis of asthma includes type 2 inflammation, which is mediated by T helper 2 (T_H_2) cells, type 2 innate lymphoid cells (ILC2s), eosinophils, and mast cells [16]. T_H_2 cells and ILC2s secrete type 2 cytokines such as interleukin (IL)-4, IL-5, and IL-13. IL-4 and IL-13 promote class switch-recombination to IgE in B cells, leading to mast cell-mediated allergic inflammation [17]. IL-5 has a role in eosinophil recruitment and activation [18]. Moreover, IL-13 is a potent inducer of mucus hyperproduction and airway remodeling [19, 20]. The production of type 2 cytokines by ILC2s are stimulated by IL-33 and IL-25, which are secreted from airway epithelial cells in response to allergens and other stimuli [16, 21, 22]. In the initial immune response of COPD, cigarette smoke and other irritants stimulate the airway epithelial cells and alveolar macrophages to secrete pro-inflammatory cytokines/chemokines such as tumor necrosis factor (TNF)-α, IL-1β, IL-6, CXCL8, and granulocyte macrophage colony-stimulating factor (GM-CSF), which recruit and activate IL-17A-secreting helper T (T_H_17) cells [23]. T_H_17 cells play an important role in COPD by promoting the production of neutrophil chemoattractants such as CXCL1 and CXCL8 in the bronchiolar epithelium [23]. Neutrophils release neutrophil elastase and other serine proteases, which results in tissue damage and mucus hypersecretion [24]. Despite significant advances in the identification of numerous inflammatory cells and mediators of airway inflammation, how immune response networks drive the onset and/or progression of each COPD and asthma symptom is not fully understood. Several animal models have been developed to investigate airway inflammatory responses during COPD (e.g. cigarette smoke exposure, elastase instillation, and targeted gene disruption) and allergic asthma (allergen sensitization and challenge) [25, 26]. Although no animal model can fully mirror human disease conditions due to the complexity and multiplicity of pathogenesis, different types of animal models provide valuable insights into the biological and clinical aspects of specific disease features.

In this study, to clarify the precise biological roles of ADGRF5 in the lung, we extended the phenotypic analysis of *Adgrf5*^*−/−*^ mice. We found that *Adgrf5*^*−/−*^ mice exhibit abnormal histological and physiological features in the lungs, resembling those of airway inflammation, and investigated time-course expression profiles of genes/proteins related to this disorder. We also provide evidence that lung ECs are another source of CCL2 in neonatal *Adgrf5*^*−/−*^ mice.

## Methods

### Mice

*Adgrf5*^*−/−*^ mice in a C57BL/6 J background were described previously [10]. In brief, *Adgrf5*^*−/−*^ mice were generated by replacing the start codon-containing exon with the *LacZ* and neomycin resistance cassette. C57BL/6 J mice were used as wildtype (WT) mice. Mice were anesthetized by the inhalation of 2.5% isoflurane in air. The animal protocols and procedures were approved by the Institutional Animal Care and Use Committee of the Tokyo Institute of Technology.

### Preparation of frozen lung sections

Mice were anesthetized by isoflurane inhalation, and the distal aorta was cut to exsanguinate the animal. The mice were perfused via the right ventricle with ice-cold phosphate-buffered saline (PBS) followed by 4% paraformaldehyde in PBS. The lungs were then separated into lobes and immersed in PBS containing 10% sucrose at 4 °C. The sucrose density was consecutively changed to 16, 18, and 20%. The lungs were then rinsed with PBS containing 50% Tissue-Tek OCT compound (Sakura Finetek, Tokyo, Japan) and frozen in Tissue-Tek OCT compound at − 80 °C. Frozen sections were cut using a cryostat (to a thickness of 7 μm) at − 20 °C.

### Alcian blue staining

Frozen lung sections were rinsed with 3% acetic acid and then stained with Alcian blue solution (pH 2.5, Muto Pure Chemicals, Tokyo, Japan) for 20 min at room temperature (approximately 25 °C). After washing with water for 1 min, tissues were incubated with 3% acetic acid for 3 min, washed with water for 1 min, and nuclei were stained with hematoxylin (Muto Pure Chemicals) for 2 min. After washing with water for 10 min, the sections were dehydrated using a graded ethanol series (70, 80 90, 95, and 100%), defatted with xylene twice, and mounted in Malinol (Muto Pure Chemicals).

### Immunohistochemistry

Frozen lung sections were permeabilized with 0.1% triton X-100 in PBS for 10 min and then blocked with 1% bovine serum albumin (BSA; Sigma-Aldrich, St. Louis, MO, USA) in PBS for 30 min. The sections were stained with a primary antibody at a dilution of 1:1000 for 1 h followed by incubation with an Alexa Fluor 488-conjugated anti-mouse or rabbit IgG antibody (Thermo Fisher Scientific, Waltham, MA, USA) for 1 h. The following primary antibodies were used: anti-MUC5AC (45 M1, Thermo Fisher Scientific), anti-SAM-pointed domain-containing Ets-like factor (SPDEF; LifeSpan BioSciences, Seattle, WA, USA), and anti-Forkhead box A2 (FOXA2; Abcam, Cambridge, UK). The nuclei were stained with Hoechst 33342 (5 μg/ml; Thermo Fisher Scientific) for 30 min. Signals were captured with a laser scanning confocal microscope (LSM780, Carl Zeiss, Oberkochen, Germany) and analyzed using Zen software (Carl Zeiss).

### Giemsa staining of cells in bronchoalveolar lavage (BAL) fluid

BAL was performed as described previously [10]. In brief, mice were exsanguinated by cutting the abdominal aorta. A 1.0-ml syringe connected to a 26-gauge blunt needle was inserted into the trachea and 0.5–0.7 ml of PBS was injected and aspirated through the needle three times. BAL fluid was centrifuged at 1000×*g* for 5 min and the resulting cell pellet was resuspended in fetal bovine serum. Cells were smeared on a glass slide, fixed with methanol for 5 min, and incubated with 1 ml of Wright-Giemsa’s stain solution (Muto Pure Chemicals) for 2 min. The cells were then added to 1 ml of M/15 phosphate buffer solution, pH 6.4 (Muto Pure Chemicals) and incubated for 12 min. After washing with water for 1 min, samples were dried using a dryer and mounted in Malinol. The images of stained cells were captured with a TOCO digital slide scanner (Claro, Hirosaki, Japan). Cells were identified using standard morphological criteria.

### Staining of eosinophils in lung sections

Staining of eosinophils was performed with an Eosinophil-Mast Cell Stain Kit (ScyTek Laboratories, Logan, UT, USA). Frozen lung sections were incubated with Astra Blue Solution for 30 min followed by Vital New Red Solution for 30 min. After washing with water, the sections were counterstained with Mayer’s hematoxylin (Lille’s modification) for 15 s. Samples were washed with water at 42 °C for 30 min, dehydrated, and then mounted in Malinol.

### In situ hybridization

The lungs of WT mice (8-weeks-old) were inflation-fixed at 26-cm H_2_O pressure with 4% paraformaldehyde in 0.1 M phosphate buffer (pH 7.4) for 5 min, excised, post-fixed in the same fixative overnight at 4 °C, and then paraffin sections were prepared (6 μm thick). The sections were hybridized with digoxigenin-labeled RNA probes corresponding to nucleotide positions 30–1563 of mouse *Adgrf5* (Genbank accession number NM_001081178.1) as described previously [27]. The sections were counterstained with Kernechtrot (Muto Pure Chemicals).

### Masson’s trichrome staining

Frozen lung sections were incubated with mordant containing 50 mg/ml potassium bichromate and 50 mg/ml trichloroacetic acid (Muto Pure Chemicals) for 20 min. After washing with water for 1 min, they were incubated with Weigert’s iron hematoxylin solution (Muto Pure Chemicals) for 3 min, washed with water for 10 min, and then incubated with mordant containing 2.5% phosphomolybdic acid and 2.5% phosphotungstic acid (Muto Pure Chemicals) for 30 s. After washing with water for 1 min followed by 1% acetic acid for 10 s, the sections were incubated with 0.75% Orange G solution (Muto Pure Chemicals) for 5 min, washed with 1% acetic acid for 10 s, and then stained with 12 mg/ml Ponceau xylidine and 8 mg/ml fuchsin S solution (Muto Pure Chemicals) for 30 min. After washing with 1% acetic acid for 10 s, the sections were incubated with 2.5% phosphotungstic acid (Muto Pure Chemicals) followed by Aniline Blue solution (Muto Pure Chemicals) for 3 min. After washing with 1% acetic acid for 10 s, the sections were dehydrated with a graded ethanol series (70, 80 90, 95, and 100%), defatted with xylene twice, and mounted in Malinol.

### Quantification of the amount of collagen in lung sections

Amounts of total collagen and total non-collagenous proteins were determined with a Sirius red/Fast green collagen staining kit (Chondrex, Redmond, WA, USA). Frozen lung sections were stained with Sirius red and Fast green (Dye solution) for 30 min at room temperature. After washing with distilled water, both dyes were eluted from the sections with Dye Extraction Buffer. The absorbance of the eluted dye solutions was measured at 540 nm (Sirius red) and 605 nm (Fast green). Levels of fibrillar collagens and non-collagenous proteins in the sections were calculated according to the manufacturer’s instructions.

### Quantitative real-time PCR (qPCR)

Total RNA from whole lungs, post-lavage lungs, or primary ECs was isolated using Isogen II (Nippon Gene, Tokyo, Japan) according to the manufacturer’s instructions. Single-stranded cDNA was prepared from 0.05–5 μg of total RNA with oligo(dT) primers and SuperScript III reverse transcriptase (Thermo Fisher Scientific) according to the manufacturer’s instructions, and was used as a template for qPCR amplification. Amplification was performed on a Thermal Cycler Dice Real Time System (Takara, Shiga, Japan) using SYBR Premix ExTaq II (Tli RNase H Plus) (Takara) according to the manufacturer’s instructions. The primer sets used were as follows: *Tgfb1*, 5′-cacgtggaaatcaacgggatcag-3′ and 5′-cgcacacagcagttcttctctg-3′; *Col1a1*, 5′-acctacagcacccttgtggac-3′ and 5′-agggagccacatcgatgatgg-3′; *Fn1*, 5′-tggcagtggtcatttcagatgc-3′ and 5′-ttcccatcgtcatagcacgttg-3′; *Tnc*, 5′-aaccacagtcagggcgttaac-3′ and 5′-atttcggaagttgctgggtctc-3′; *Muc5ac*, 5′-gaaagttggtcccattctgg-3′ and 5′-cggtgttcatggtacgatttc-3′; *Muc5b*, 5′-ccttgccacttccactacga-3′ and 5′-gagcacggaggtacagttatcca-3′; *Slc26a4*, 5′-catcatctccggagttagcac-3′ and 5′-cgaacacaaaatacgtcaggatag-3′; *Clca1*, 5′-atccacaccaaaacgagaaggc-3′ and 5′-tgcttcggagattgcatcgttg-3′; *Il4*, 5′-caaacgtcctcacagcaacg-3′ and 5′-tgcagctccatgagaacactag-3′; *Il5*, 5′-agcaatgagacgatgaggcttc-3′ and 5′-cccacggacagtttgattcttcag-3′; *Il13*, 5′-aagatctgtgtctctccctctgac-3′ and 5′-ataccatgctgccgttgcac-3′; *Ccl2*, 5′-gaagccagctctctcttcctc-3′ and 5′-ttgctggtgaatgagtagcag-3′; *S100a8*, 5′-tgagtgtcctcagtttgtgcag-3′ and 5′-tgccacacccacttttatcacc-3′; *S100a9*, 5′-aaatggtggaagcacagttggc-3′ and 5′-tgggttgttctcatgcagcttc-3′; *Saa3*, 5′-cattctttgcatcttgatcctg-3′ and 5′-cgagcatggaagtatttgtctg-3′; *Gapdh*, 5′-aggtcggtgtgaacggatt-3′ and 5′-tgccgtgagtggagtcatac-3′.

### Antibody array

Post-lavage lungs, prepared from three mice (10-weeks-old), were homogenized in PBS containing 1% triton X-100 and protease inhibitors (5 mg/ml aprotinin, 10 mM leupeptin, 1 mM pepstatin, and 1 mM phenylmethylsulfonyl fluoride). The homogenates were centrifuged for 20 min at 18,340×*g* at 4 °C. The resulting supernatants (200 μg of proteins) were used with a Proteome Profiler Array (Mouse cytokine array panel A; R&D Systems, Minneapolis, MN, USA) according to the manufacturer’s instructions. Chemiluminescence signals on the antibody array were detected using an ImageQuant LAS 4000 image analyzer (GE Healthcare, Little Chalfont, UK).

### Magnetic isolation of lung ECs

Lung tissue was excised from an anesthetized mouse (1-week-old), minced, and then incubated in Dulbecco’s Modified Eagle’s Medium (DMEM; Nacalai Tesque, Kyoto, Japan) containing 1 mg/ml collagenase/dispase solution (Roche, Basel, Switzerland) and 5 U/ml DNase I (Roche) for 45 min at 37 °C. The digested pieces were further minced by passing them through a 20-gauge needle and then filtered with a 70-μm cell strainer (BD Biosciences, San Jose, CA, USA). The filtrate was centrifuged for 5 min at 400×*g* at 20 °C, and the resulting cell pellet was suspended in PBS containing 0.1% BSA. The cells were incubated with Dynabeads (Invitrogen, Carlsbad, CA, USA) precoated with rat anti-mouse CD31 antibody (BD Biosciences) for 30 min at room temperature. ECs bound to the Dynabeads were collected with a magnet, washed using PBS with 0.1% BSA, and then cultured in a 60-mm dish coated with 2% gelatin in Endothelial Cell Growth Medium 2 (Takara). The purity of isolated ECs was > 95%, which was confirmed by immunofluorescence microscopy using anti-CD31 and anti-CD102 antibodies (BD Biosciences).

### Western blot analysis

Whole lungs were homogenized in PBS containing 1% triton X-100 and protease inhibitors (5 mg/ml aprotinin, 10 mM leupeptin, 1 mM pepstatin, and 1 mM phenylmethylsulfonyl fluoride). After clarifying by centrifugation at 10,000×*g* for 30 min, the lysates (20 or 30 μg of proteins) were subjected to western blot analysis as described previously [28]. The following primary antibodies were used: anti-ADGRF5 (N7), 1:1000 [2]; anti-SPDEF, 1:1000; anti-transforming growth factor (TGF)-β1 (Novus Biologicals, Littleton, CO, USA), 1:1000; anti-IL-33 (396,118, R&D Systems), 1:1000; anti-IL-25 (68C1039.2, Novus Biologicals), 1:500; anti-TPSAB1 (AA1, Bio-Rad, Hercules, CA, USA), 1:1000; anti-CCL2 (2D8, Novus Biologicals); anti-α tubulin (Sigma-Aldrich), 1:8000.

### Blood gas and urine pH measurements

Arterial blood was drawn from the left ventricle of anesthetized adult mice (12-weeks-old) with a 26-gauge needle fitted on a heparin-coated syringe (364,356, Becton-Dickinson, Oxford, UK). Blood gas status was determined using a blood gas analyzer (ABL 505, Radiometer, Copenhagen, Denmark). For urine pH measurements, mice at 10 weeks of age were placed in metabolic cages for 1 day, and then 12-h urine samples were collected. Urine pH was measured with a glass electrode from Koto-Biken Medical Laboratories (Tokyo, Japan).

### Measurement of serum IgE concentrations

Blood was taken from the left ventricle of an anesthetized mouse using a 24-gauge needle. Serum IgE levels were determined by enzyme-linked immunosorbent assay (ELISA) with a commercial kit (Yamasa EIA, Yamasa, Chiba, Japan) according to the manufacturer’s instruction.

### Administration of a C-C chemokine receptor type 2 (CCR2) antagonist to mice

RS504393 (Cayman Chemical, Ann Arbor, MI, USA) was dissolved in dimethylformamide at 10 mg/ml and then diluted to 0.4 mg/ml with a 0.9% sterile NaCl solution. The RS504393 solution was filtered through a 0.22-μm filter (Millipore, Burlington, MA, USA) and injected subcutaneously into the backs of anesthetized *Adgrf5*^*−/−*^ mice (2-weeks-old) at 2 mg/kg body weight, once daily for 8 days. The control group was treated with an equal volume of vehicle using the same protocol. The mice were sacrificed 24 h after the last injection.

### Cell culture

NCI-H292 cells were obtained from ECACC (Porton Down, UK) and cultured in RPMI-1640 medium (Nacalai Tesque) containing 10% fetal bovine serum, penicillin (100 U/ml), and streptomycin (100 μg/ml) at 37 °C. Cells at 80% confluency were treated with or without recombinant human CCL2 (100 ng/ml, Peprotech, Rocky Hill, NJ, USA) for 18 h at 37 °C. qPCR was performed with the following primer sets: *MUC5AC*, 5′-tggggacagctcttccatgtac-3′ and 5′-tgcagtgcagggtcacattc-3′; *SLC26A4*, 5′-tttcctggacgttgttggagtg-3′ and 5′-tgtcgtcaaagaacccgcattg-3′; *GAPDH*, 5′-tgcaccaccaactgcttagc-3′ and 5′-atggcatggactgtggtcatg-3′.

### Image and statistical analysis

Light microscopic images were captured with a TOCO digital slide scanner or an Axioskop microscope (Zeiss) equipped with an AxioCam HRc color microscope camera (Zeiss). Quantification of the stained area, basement membrane length, and band intensity was performed using ImageJ software (National Institute of Health, Bethesda, MD, USA). For Alcian blue- and Masson’s trichrome-stained samples, the blue-stained regions were selected using the magic wand tool in PhotoShop CS5 software (Adobe Systems, San Jose, CA, USA) and converted to black with ImageJ software. The pixel area of the black region was quantified with ImageJ software. The volume of epithelial mucus was calculated by multiplying the blue stained area by the thickness of the section. Data are presented as the mean ± SEM of at least three independent experiments. A student’s *t* test or one-way ANOVA followed by a Tukey’s *post-hoc* test was performed for the statistical analysis of data using GraphPad Prism 5 software (GraphPad Software, San Diego, CA, USA). A value of *p* < 0.05 was considered significant.

## Results

### Mucus hyperproduction in the lungs of *Adgrf5*^−/−^ mice

**Fig. 1:**
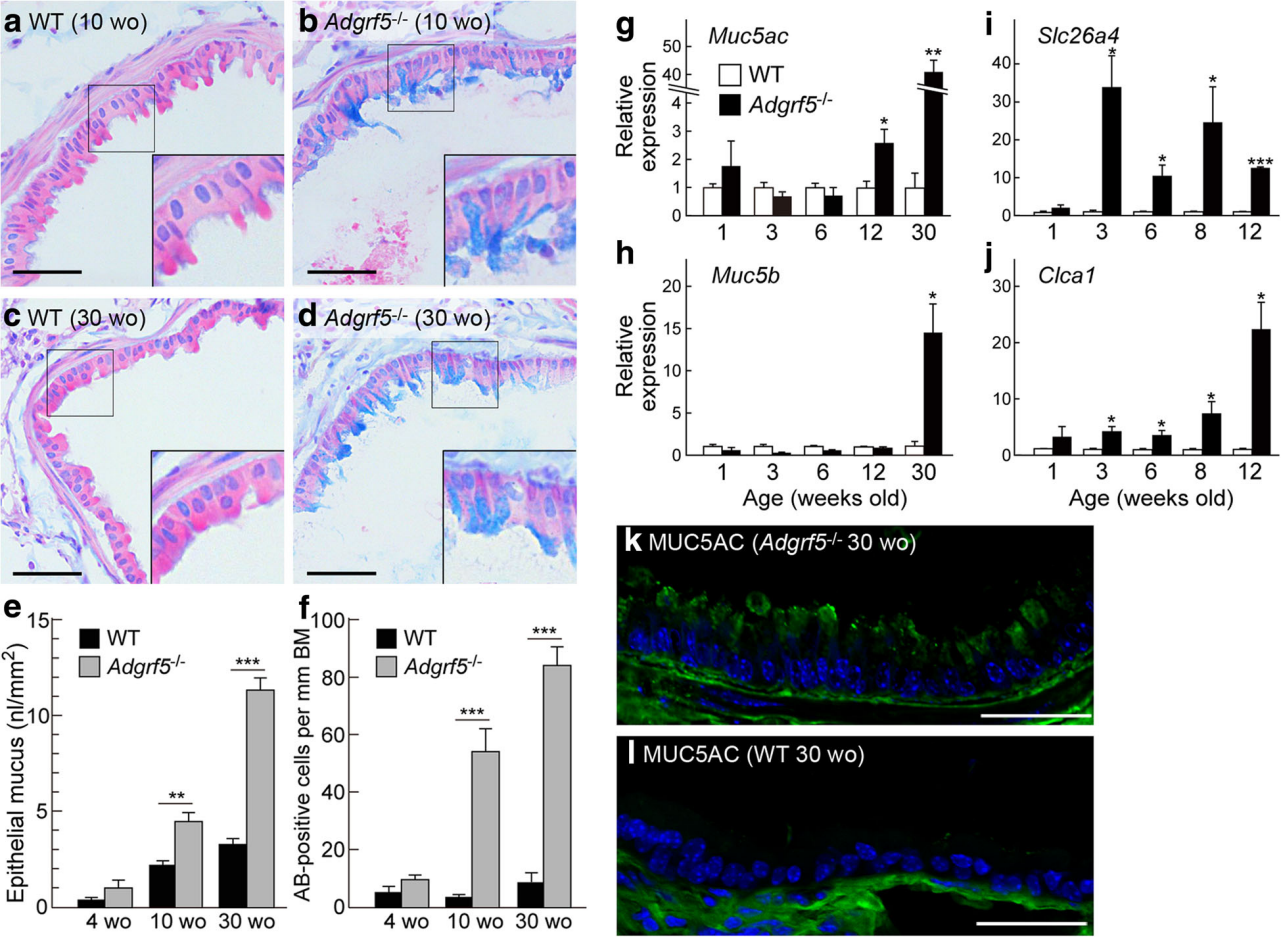
Mucus hyperproduction in the bronchiolar epithelium of *Adgrf5*^*−/−*^ mice. **a**–**d** Alcian blue staining was performed on lung sections from WT (**a** and **c**) and *Adgrf5*^*−/−*^ mice (**b** and **d**) at 10 (**a** and **b**) and 30 (**c** and **d**) weeks of age. Mucus was stained blue. Nuclei (blue purple) and cytoplasm (pink) were counter-stained by hematoxylin and eosin. Insets show high magnification images of areas indicated by squares. Scale bars, 50 μm. **e** The volume of Alcian blue-positive mucosubstance in the epithelium was estimated from more than six randomly-selected bronchioles per mouse. Results are expressed as the volume of mucosubstance (nl) per surface area (mm^2^) of the bronchiole basement membrane, and are presented as the mean ± SEM (*n* = 5). **p < 0.01 and ***p < 0.0005. **f** The density of Alcian blue (AB)-positive cells was evaluated in more than six randomly selected bronchioles per mouse. Results are expressed as the number of Alcian blue-positive cells per millimeter length of bronchial basement membrane (BM), and are presented as mean ± SEM (*n* = 5). ***p < 0.0005. **g**–**j** The mRNA expression of *Muc5ac* (**g**), *Muc5b* (**h**), *Slc26a4* (**i**), and *Clca1* (**j**) was analyzed by qPCR in whole (1 week old) or post-lavage (3, 6, 8, 12, and 30 weeks of age) lungs from WT and *Adgrf5*^*−/−*^ mice. The data were normalized to *Gapdh* levels and expressed as values relative to corresponding WT mice. Values are presented as the mean ± SEM (*n* = 3–4). *p < 0.05; **p < 0.01; ***p < 0.001. **k** and **l** Immunofluorescence confocal microscopy was performed on lung sections from *Adgrf5*^*−/−*^ (**k**) and WT mice (**l**) at 30 weeks of age with an antibody specific for MUC5AC (green). The nuclei were stained with Hoechst 33342 (blue). Scale bars, 40 μm

In our previous study, we found that targeted disruption of ADGRF5 in mice causes alveolar inflammation accompanied by the accumulation of activated pulmonary macrophages [12]. In this study, to further investigate lung inflammation in *Adgrf5*^*−/−*^ mice, we first examined bronchial inflammation in these animals by Alcian blue staining using lung sections from WT and *Adgrf5*^*−/−*^ mice at 10 and 30 weeks of age. A marked deposition of mucoid material was observed in the bronchiolar epithelium of *Adgrf5*^*−/−*^ mice at both ages (Fig. 1b, d vs. a, c). Quantification revealed that the volume of epithelial mucus and the number of Alcian-blue-positive cells were increased gradually with age in *Adgrf5*^*−/−*^ mice; however, no such increase was observed in *Adgrf5*^*−/−*^ mice at 4 weeks of age (Fig. 1e, f). These results suggest that mucus hyperproduction occurs in the bronchioles of *Adgrf5*^*−/−*^ mice.

### Increased expression of airway mucin genes and related mediators of mucus production in *Adgrf5*^−/−^ lungs

We next determined the mRNA expression levels of lung parenchymal *Muc5ac* and *Muc5b*, which encode the major gel-forming mucins found in airway mucus [29]. qPCR was performed using total RNA isolated from whole lungs of 1-week-old mice or post-lavage lungs of 3-, 6-, 12-, and 30-week-old mice. There was no significant difference in the expression of the two mucin genes between WT and *Adgrf5*^*−/−*^ lung samples from 1-, 3-, and 6-week-old mice (Fig. 1g, h). However, at 12 weeks of age, *Muc5ac* expression was increased 2.6-fold (*p* < 0.05) in *Adgrf5*^*−/−*^ lungs compared to that in WT lungs, whereas there was no change in *Muc5b* expression (Fig. 1g, h). At 30 weeks of age, both mucin genes were upregulated in *Adgrf5*^*−/−*^ lungs compared to expression in WT lungs (41.4-fold, *p* < 0.01 for *Muc5ac* and 14.5-fold, *p* < 0.05 for *Muc5b*; Fig. 1g, h). To confirm the increased *Muc5ac* expression at the protein level, immunohistochemistry was performed using lung cryo-sections from 30-week-old WT and *Adgrf5*^*−/−*^ mice. Evident MUC5AC staining was observed in the apical region of the bronchiolar epithelial cells of *Adgrf5*^*−/−*^ mice (Fig. 1k), whereas this was diminished in the bronchioles of WT mice (Fig. 1l). Mucus hyperproduction and increased MUC5AC expression are characteristic features of asthma and COPD [30]. Further, SLC26A4 and CLCA1 are known to be involved in mucus production and allergic inflammation in this disease [31–33]. mRNA expression of these markers was upregulated in *Adgrf5*^*−/−*^ lungs compared to that in WT lungs at 3 weeks of age (33.6-fold, *p* < 0.05 for *Slc26a4* and 4.3-fold, *p* < 0.05 for *Clca1*), whereas no such significant differences were observed at 1 week of age (Fig. 1i, j). In addition, a > 10-fold increase in the expression of *Slc26a4* was detected in *Adgrf5*^*−/−*^ lungs at 6, 8, and 12 weeks of age (Fig. 1i). In contrast, higher expression of *Clca1* became apparent in *Adgrf5*^*−/−*^ lungs from 8 weeks of age (7.4-fold increase, *p* < 0.05; Fig. 1j). These results suggest that mucus production in *Adgrf5*^*−/−*^ lungs is stimulated between 6 and 12 weeks of age, and becomes excessive at 30 weeks, supporting the above histological observation of age-dependent mucus overproduction.

### Increased expression of SPDEF and decreased expression of FOXA2 in the bronchiolar epithelium of *Adgrf5*^−/−^ mice

**Fig. 2:**
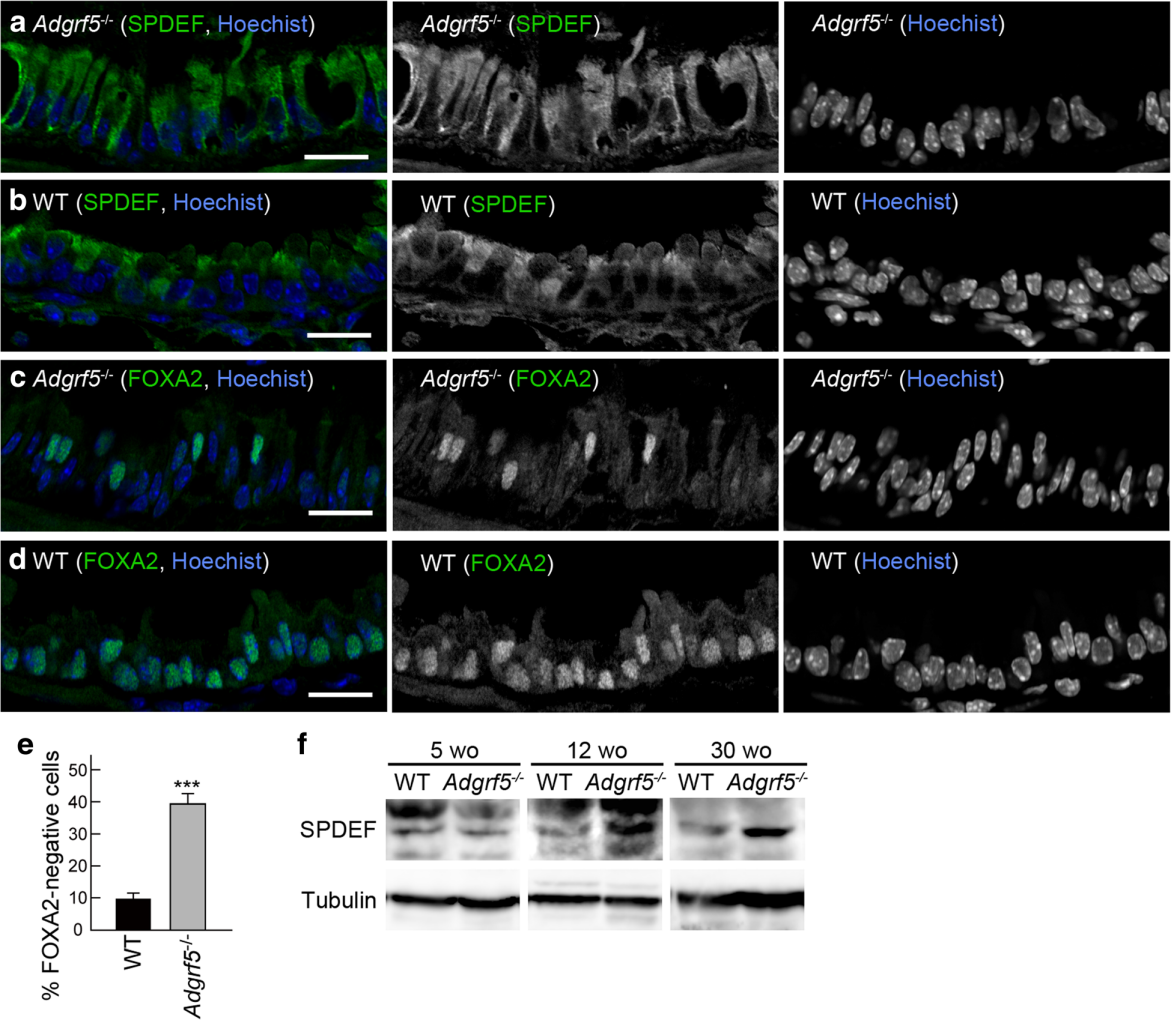
Increased expression of SPDEF and decreased expression of FOXA2 in the bronchiolar epithelium of *Adgrf5*^−/−^ mice. **a**–**d** Immunofluorescence confocal microscopy was performed on lung sections from *Adgrf5*^*−/−*^ (**a** and **c**) and WT mice (**b** and **d**) at 30 weeks of age with an antibody specific for SPDEF (**a** and **b**, green in left panels, middle panels) or FOXA2 (**c** and **d**, green in left panels, middle panels). The nuclei were stained with Hoechst 33342 (blue in left panels, right panels). Scale bars, 20 μm. **e** FOXA2-negative bronchiolar epithelial cells and total bronchiolar epithelial cells were counted (total number of cells counted > 800). Results are expressed as percentage of FOXA2-negative cells relative to total bronchiolar epithelial cells, and are presented as the mean ± SEM (*n* = 6). ***p < 0.0001. **f** Whole lung lysates (20 μg of protein) from WT and *Adgrf5*^*−/−*^ mice (5, 12, and 30 weeks of age) were analyzed by western blotting using antibodies specific for SPDEF (top) and α-tubulin (bottom)

The increased number of Alcian-blue-positive cells led us to examine whether mucous cell metaplasia occurs in the bronchiolar epithelium of *Adgrf5*^*−/−*^ mice. SPDEF is a key player in a transcriptional network that mediates mucous cell metaplasia [34]. One mechanism associated with the function of SPDEF is the suppression of FOXA2, a potent inhibitor of MUC5AC expression and mucous cell metaplasia [35, 36]. Therefore, we analyzed SPDEF and FOXA2 expression by immunohistochemistry using lung cryo-sections from 30-week-old WT and *Adgrf5*^*−/−*^ mice. SPDEF expression was abundant in the bronchiolar epithelial cells of *Adgrf5*^*−/−*^ mice (Fig. 2a), whereas it was less abundant in those of WT mice (Fig. 2b). As expected, nuclear expression of FOXA2 in the epithelial cells was reduced in *Adgrf5*^*−/−*^ mice compared to that in WT mice (Fig. 2c, d); moreover, the FOXA2-negative cell population significantly increased from 9.8% in WT mice to 39.3% in *Adgrf5*^*−/−*^ mice (*p* < 0.0001; Fig. 2e). In addition, western blot analysis of whole lung lysates showed that SPDEF expression was increased at 12 and 30 weeks, but not at 5 weeks of age (Fig. 2f). These results suggest that mucous cell metaplasia occurs in the bronchioles of *Adgrf5*^*−/−*^ mice.

### Adgrf5 is not expressed in the bronchiolar epithelium

**Fig. 3:**
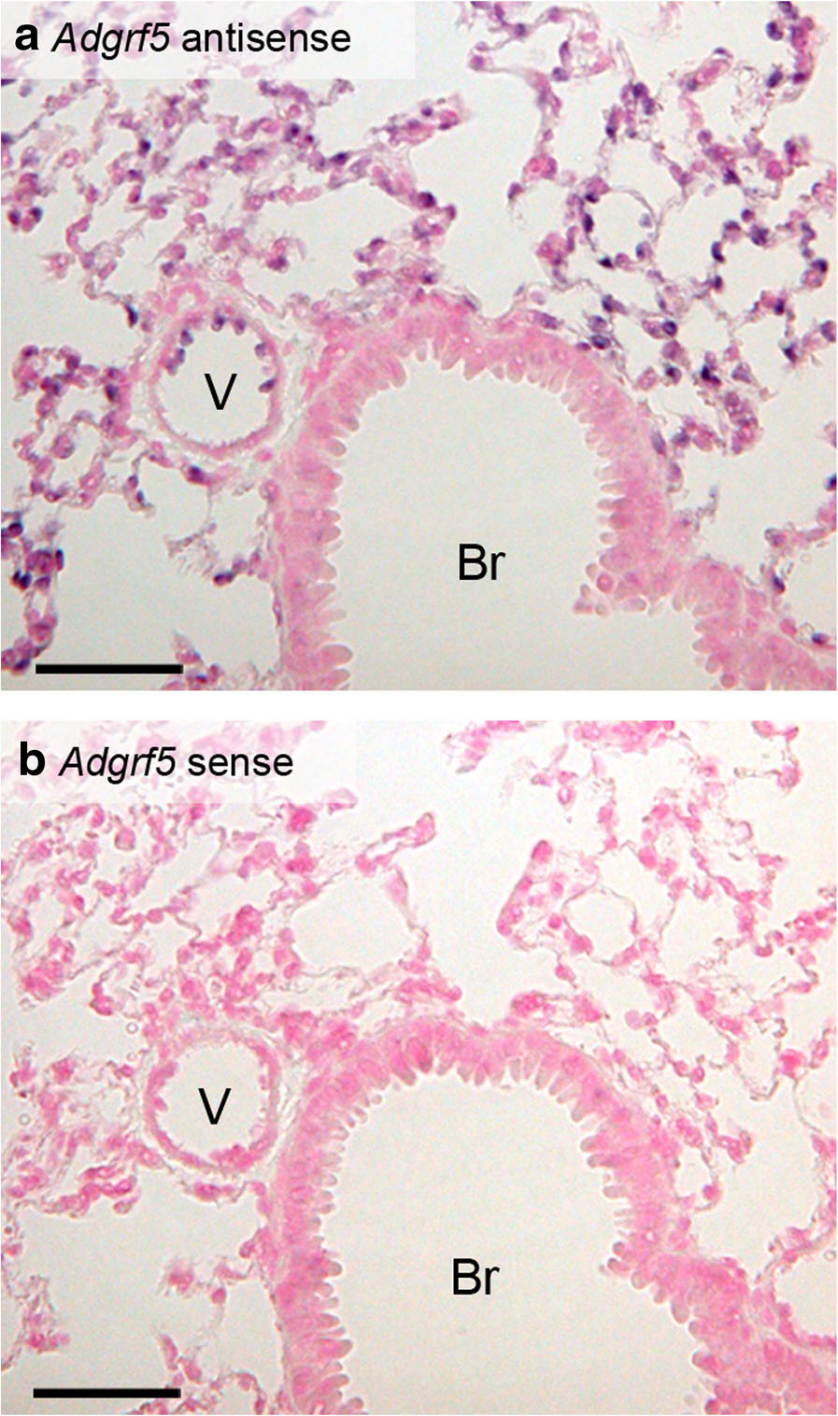
*Adgrf5* is not expressed in the bronchial epithelium. In situ hybridization was performed on paraffin-embedded lung sections from WT mice (8-weeks-old) using an *Adgrf5*-specific antisense probe (**a**) or a corresponding sense probe (**b**). The sections were counterstained with Kernechtrot (pink). Br, bronchiole and V, blood vessel. Scale bars, 50 μm

The bronchial abnormalities in *Adgrf5*^*−/−*^ mice led us to examine the expression of ADGRF5 in the bronchiolar epithelium. In situ hybridization histochemistry was performed on lung sections from 8-week-old WT mice. Positive signals were detected with the *Adgrf5* antisense probe in alveolar cells and blood vessel ECs (Fig. 3a). However, there was no signal in the bronchiolar epithelium (Fig. 3a, compared to Fig. 3b, which was obtained with the corresponding sense probe). This result is consistent with the previous reports demonstrating undetectable or very limited expression of ADGRF5 in airway epithelia [8, 10]. Thus, the observed mucous cell metaplasia and mucus hyperproduction are most likely secondary effects of ADGRF5 disruption.

### Subepithelial fibrosis in the *Adgrf5*^−/−^ lung

**Fig. 4:**
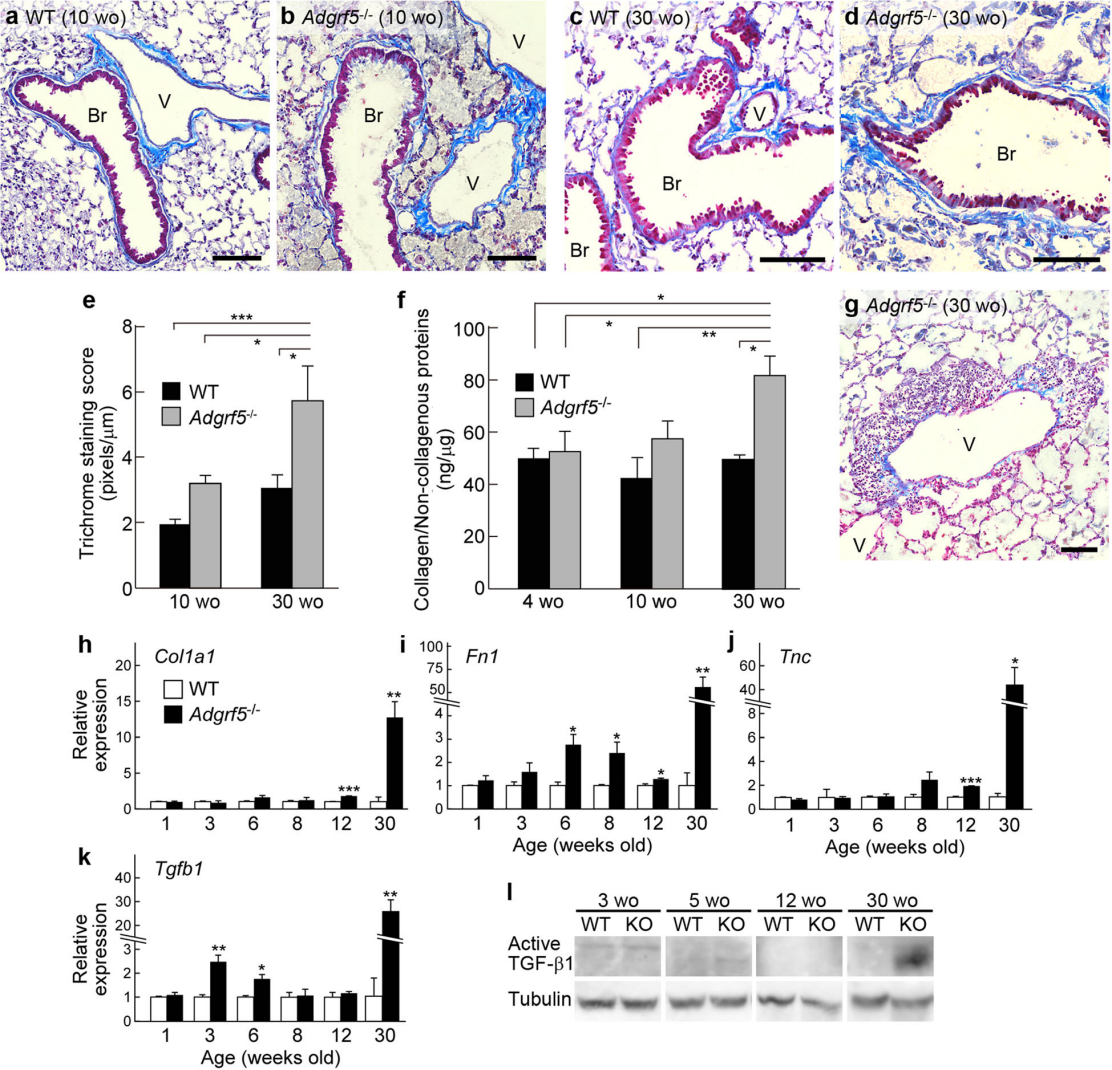
Subepithelial fibrosis in the lungs of aged *Adgrf5*^*−/−*^ mice. **a**–**d** and **g** Masson’s trichrome staining was performed on lung sections from WT (**a** and **c**) and *Adgrf5*^*−/−*^ mice (**b**, **d**, and **g**) at 10 (**a** and **b**) and 30 (**c**, **d**, and **g**) weeks of age. Fibrous collagen was stained blue, and nuclei and cytoplasm were stained red. Br, bronchiole and V, blood vessel. Scale bars, 100 μm. **e** Trichrome-stained areas were evaluated in more than six randomly-selected bronchioles per mouse. Results are expressed as the area of trichrome staining (pixels) per micrometer length of bronchiole basement membrane, and are presented as the mean ± SEM (*n* = 3). **p < 0.01 and ***p < 0.001. **f** The amounts of fibrillar collagens and non-collagenous proteins in lung sections from WT and *Adgrf5*^*−/−*^ mice (4, 10, and 30 weeks of age) were estimated using a Sirius red/Fast green collagen staining kit. Results are expressed as the amount of fibrillar collagens (ng) relative to non-collagenous protein levels (μg), and are presented as mean ± SEM (*n* = 3). *p < 0.05 and **p < 0.01. **h**–**k** The mRNA expression of *Col1a1* (**h**), *Fn1* (**i**), *Tnc* (**j**), and *Tgfb1* (**k**) was analyzed by qPCR in whole (1-week-old) or post-lavage (3, 6, 8, 12 and 30 weeks of age) lungs from WT and *Adgrf5*^*−/−*^ mice. The data were normalized to *Gapdh* levels and expressed as values relative to corresponding WT mice. Values are presented as the mean ± SEM (*n* = 3–4). *p < 0.05; **p < 0.01; ***p < 0.001. **l** Whole lung lysates (20 μg of protein) from WT and *Adgrf5*^*−/−*^mice (KO) (3, 5, 12, and 30 weeks of age) were analyzed by western blotting using antibodies specific for TGF-β1 (top) and α-tubulin (bottom)

Masson’s trichrome staining was performed on lung sections from 10- and 30-week-old WT and *Adgrf5*^*−/−*^ mice. Bronchial subepithelial collagen deposition was significantly increased in *Adgrf5*^*−/−*^ lungs compared to that in WT lungs at 30 weeks of age, whereas it was slightly but not significantly increased in 10-week-old *Adgrf5*^*−/−*^ lungs (Fig. 4a–e). In addition, marked inflammatory cell infiltration was observed around the blood vessels of *Adgrf5*^*−/−*^ mice at 30 weeks of age (Fig. 4g). When collagen amounts in lung sections were measured by colorimetric analysis using Sirius Red and Fast Green, collagen accumulation was detected in *Adgrf5*^*−/−*^ lungs at 30 weeks, but not at 4 and 10 weeks of age (Fig. 4f). These results suggest that fibrosis likely becomes apparent in *Adgrf5*^*−/−*^ lungs between 12 and 30 weeks of age. To verify this hypothesis, we examined mRNA expression levels of fibrosis markers including type I collagen (*Col1a1*), fibronectin (*Fn1*), tenascin C (*Tnc*), and TGF-β1 (*Tgfb1*) in whole lungs (1-week-old mice) and post-lavage lungs (3-, 6-, 8-, 12-, and 30-week-old mice) from WT and *Adgrf5*^*−/−*^ animals. There were no changes in the expression of the four fibrosis markers in 1-week-old *Adgrf5*^*−/−*^ lungs (Fig. 4h–k). The expression of *Col1a1* and *Tnc* were comparable between WT and *Adgrf5*^*−/−*^ lungs until 8 weeks of age, and showed a slight increase in *Adgrf5*^*−/−*^ lungs at 12 weeks of age (1.8-fold, *p* < 0.001 for *Col1a1*; 1.9-fold, *p* < 0.0001 for *Tnc*; Fig. 4h, j). A similar slight, but significant, upregulation was detected in *Adgrf5*^*−/−*^ lungs at 6, 8, and 12 weeks of age for *Fn1* expression (Fig. 4i) and at 3 and 6 weeks of age for *Tgfb1* expression (Fig. 4k). At 30 weeks of age, the expression all four genes was dramatically increased in *Adgrf5*^*−/−*^ lungs compared to that in WT lungs (12.7-fold, *p* < 0.01 for *Col1a1*; 78.4-fold, *p* < 0.05 for *Fn1*; 44.9-fold, *p* < 0.05 for *Tnc*; and 26.0-fold, *p* < 0.01 for *Tgfb1*; Fig. 4h–k). Furthermore, western blot analysis of whole lung lysates showed that high levels of active TGF-β1 were detected only in *Adgrf5*^*−/−*^ lungs at 30 weeks of age (Fig. 4l).

### Respiratory acidosis in *Adgrf5*^−/−^ mice

**Fig. 5:**
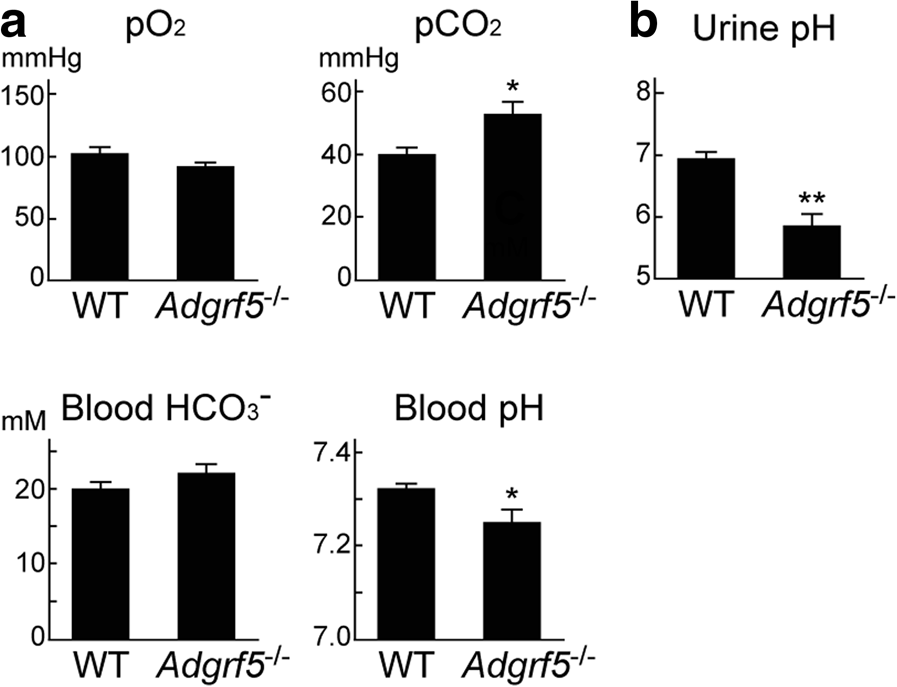
Blood gas status and urine pH in WT and *Adgrf5*^*−/−*^ mice. **a** The partial pressures of O_2_ (pO_2_) and CO_2_ (pCO_2_) and pH in arterial blood of WT and *Adgrf5*^*−/−*^ mice (12-weeks-old) were measured using a blood gas analyzer (ABL 505 Radiometer). Bicarbonate (HCO_3_^−^) concentrations were calculated from the pCO_2_ and pH data. Values are presented as the mean ± SEM (*n* = 7). *p < 0.05. **b** The pH of urine collected from WT and *Adgrf5*^*−/−*^ mice (10-weeks-old) was determined using a grass electrode. Data are presented as the mean ± SEM (*n* = 6). **p < 0.001

Chronic lung diseases such as COPD and asthma lead to respiratory acidosis due to hypoventilation [37]. *Adgrf5*^*−/−*^ mice exhibit asthma- and COPD-like signs, as well as pulmonary surfactant accumulation, which might interfere with normal respiration, thereby leading to respiratory acidosis. To examine this possibility, we measured the blood gas concentration and pH in WT and *Adgrf5*^*−/−*^ mice at 12 weeks of age. CO_2_ concentrations were significantly higher (*p* < 0.05) and pH was lower (*p* < 0.05) in *Adgrf5*^*−/−*^ mice compared to those in WT mice (Fig. 5a). HCO_3_^−^ concentration was slightly, but not significantly, increased, whereas O_2_ concentration was slightly decreased in *Adgrf5*^*−/−*^ mice (Fig. 5a). The low blood pH and hypercapnia indicate that *Adgrf5*^*−/−*^ mice present with respiratory acidosis. We also measured the pH of urine samples, and the results showed that 10-week-old *Adgrf5*^*−/−*^ mice excrete acidic urine (pH 5.8, vs. pH 6.9 for WT mice; *p* < 0.001), confirming the presence of acidosis (Fig. 5b).

### Increased expression of type-2 and epithelium-derived cytokines in *Adgrf5*^*−/−*^ mice

**Fig. 6:**
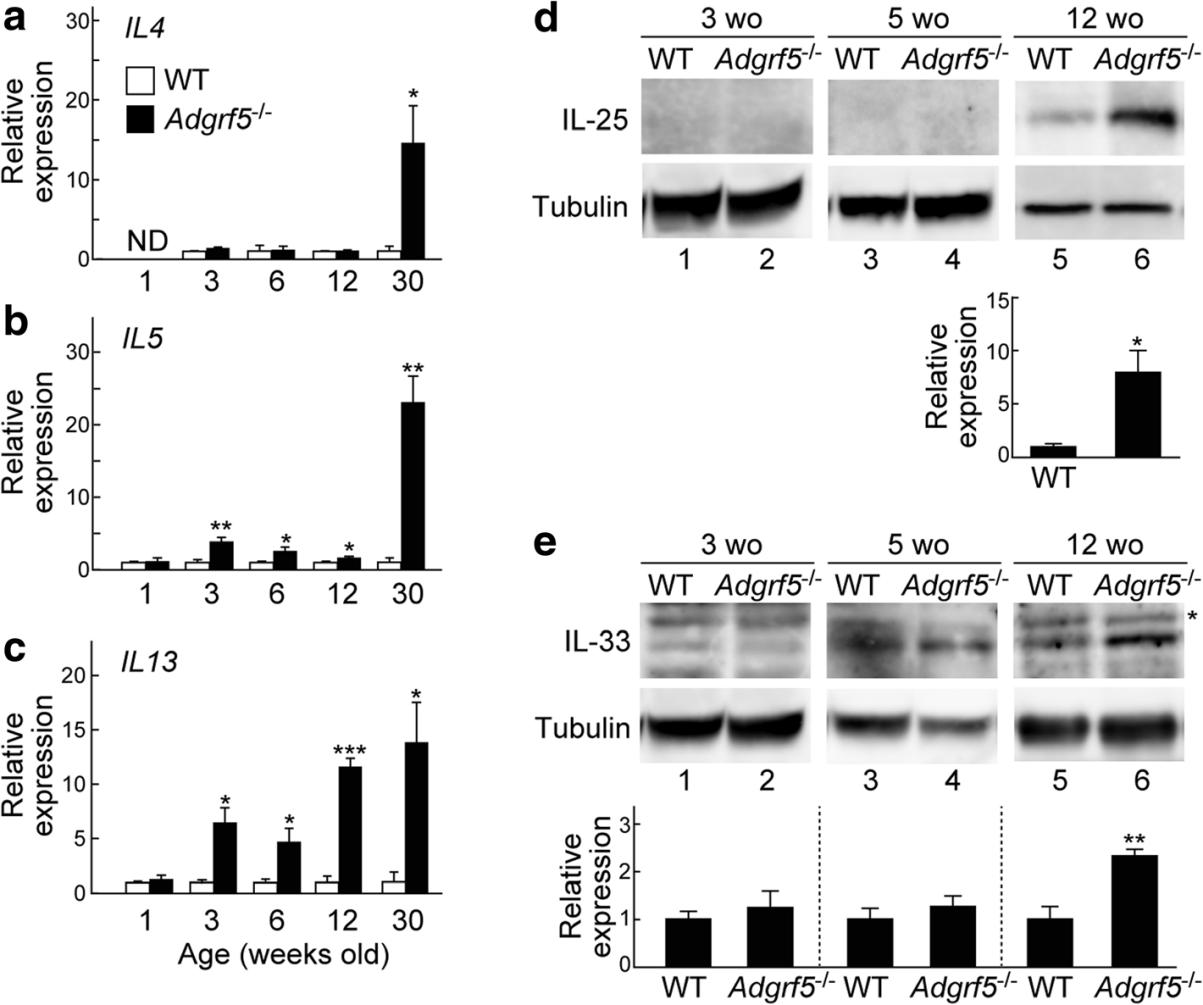
*Il4*, *Il5*, *Il13*, IL-25, and IL-33 are upregulated in the lungs of *Adgrf5*^*−/−*^ mice. **a**–**c** The mRNA expression of *Il4*, *Il5*, and *Il13* was analyzed by qPCR in whole (1-week-old) or post-lavage (3, 6, 12, and 30 weeks of age) lungs of WT and *Adgrf5*^*−/−*^ mice. The data were normalized to *Gapdh* levels and are expressed as values relative to those of corresponding WT mice. Values are presented as the mean ± SEM (*n* = 3–5). *p < 0.05; **p < 0.005; ***p < 0.0001; ND, not detected. **d** and **e** Whole lung lysates (20 μg of protein) from WT and *Adgrf5*^*−/−*^ mice (3, 5, and 12 weeks of age) were analyzed by western blotting using antibodies specific for IL-25 (**d**, top), IL-33 (**e**, top), and α-tubulin (bottom). An asterisk indicates nonspecific bands. The band intensity was normalized to that of α-tubulin and is expressed as a value relative to that in corresponding WT mice. Values are presented as the mean ± SEM (*n* = 3). *p < 0.05 and **p < 0.01

Type 2 cytokines such as IL-4, IL-5, and IL-13 play a critical role in mucous cell metaplasia and mucus hypersecretion during asthma [19, 20]. Moreover, IL-4 and IL-13 mediate the upregulation of MUC5AC, CLCA1, and SLC26A4 in the asthmatic airway epithelia [38–43]. We therefore hypothesized that mucus hyperproduction in *Adgrf5*^*−/−*^ mice might be driven by these type 2 cytokines. To examine this hypothesis, the mRNA expression of *Il4*, *Il5*, and *Il13* was analyzed in whole lungs (1-week-old mice) and post-lavage lungs (3-, 6-, 12-, and 30-week-old mice) from WT and *Adgrf5*^*−/−*^ animals. At 1 week of age, the expression of all three genes was undetectable in or comparable between WT and *Adgrf5*^*−/−*^ lungs (Fig. 6a–c). *Il4* expression in *Adgrf5*^*−/−*^ lungs was similar to that in WT lungs until 12 weeks of age (Fig. 6a). *Il5* expression in *Adgrf5*^*−/−*^ lungs was slightly, but significantly, higher than that in WT lungs at 3, 6, and 12 weeks of age (1.7–3.8-fold; Fig. 6b). *Il13* expression in *Adgrf5*^*−/−*^ lungs was moderately increased (2.6–3.8-fold) at 3 and 6 weeks of age and markedly increased, by 11.6-fold (*p* < 0.0001), compared to that in WT lungs at 12 weeks of age (Fig. 6c). At 30 weeks of age, all three genes were upregulated and highly expressed in *Adgrf5*^*−/−*^ lungs compared to expression in WT lungs (14.6-fold, *p* < 0.05 for *Il4*; 23.2-fold, *p* < 0.005 for *Il5*; 13.8-fold, *p* < 0.05 for *Il13*; Fig. 6a–c).

Next, the protein levels of IL-33 and IL-25 were compared between WT and *Adgrf5*^*−/−*^ lungs at 3, 5, and 12 weeks of age. Western blot analysis of whole lung lysates showed that both were upregulated by 2.3-fold (*p* < 0.01) and 8.0-fold (*p* < 0.05), respectively, in *Adgrf5*^*−/−*^ lungs compared to expression in WT lungs at 12 weeks of age (Fig. 6d, e, lanes 5 and 6). At 3 and 5 weeks of age, no expression of IL-25 was detected, and there was no change in the expression of IL-33 between WT and *Adgrf5*^*−/−*^ lungs (Fig. 6d, e, lanes 1–4). These results suggest that loss of ADGRF5 induces IL-33 and IL-25 expression, which is likely to promote the production of type 2 cytokines at 12 weeks of age and on.

### Increased serum IgE levels and pulmonary mast cell accumulation in *Adgrf5*^*−/−*^ mice

**Fig. 7:**
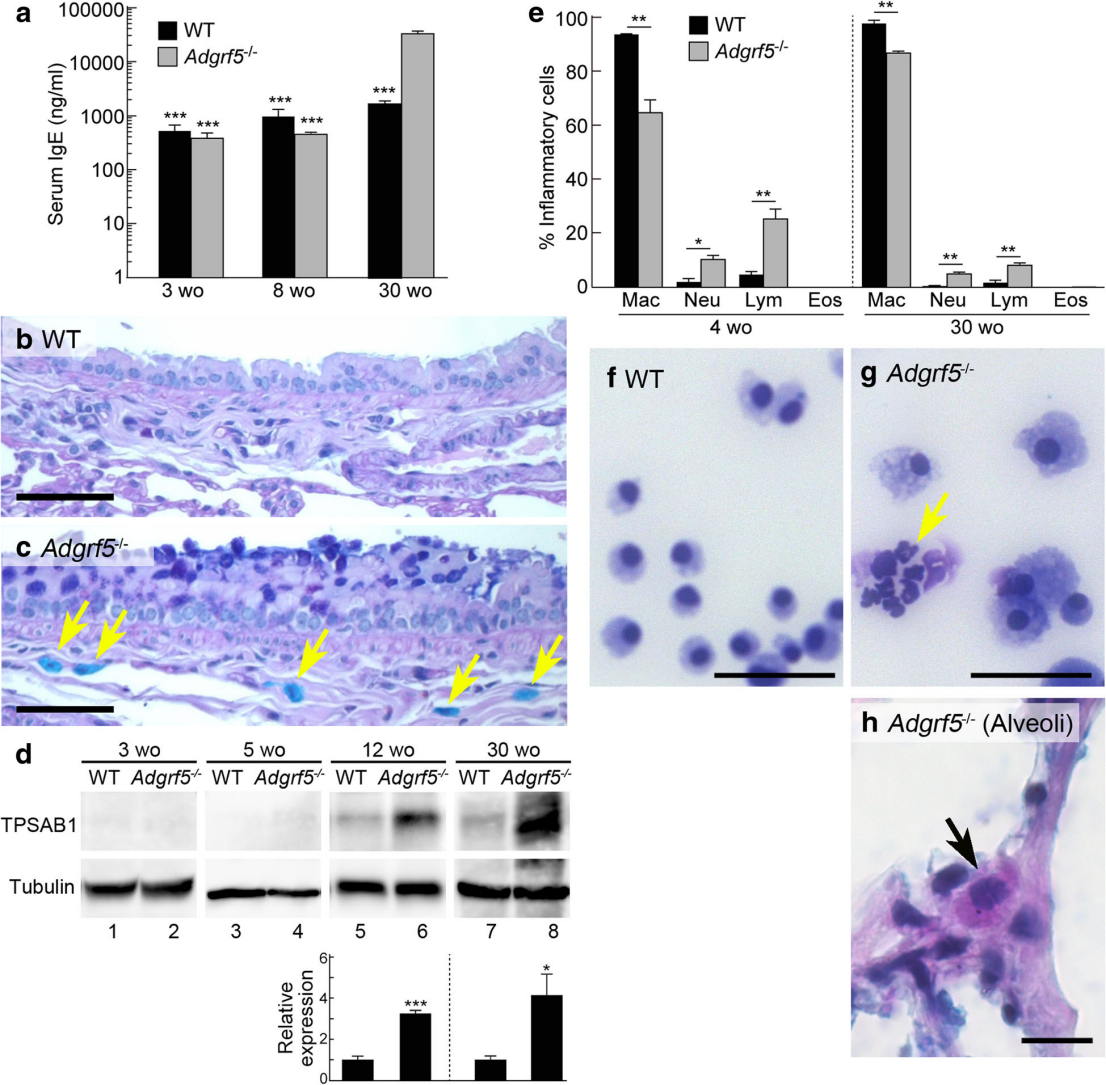
Increased serum IgE levels and pulmonary mast cell accumulation in *Adgrf5*^*−/−*^ mice. **a** The levels of total serum IgE in WT and *Adgrf5*^*−/−*^mice (3, 8, and 30 weeks of age) were determined by enzyme-linked immunosorbent assay (ELISA). Values are presented as the mean ± SEM (*n* = 3 for 3 weeks old, *n* = 5 for 8 weeks old, *n* = 4 for 30 weeks old). ***p < 0.001 vs. 30-week-old *Adgrf5*^*−/−*^ mice. **b** and **c** Alcian blue-periodic acid Schiff staining was performed on lung sections from WT (**b**) and *Adgrf5*^*−/−*^ mice (**c**) at 30 weeks of age. Nuclei (blue purple) were counter-stained with hematoxylin. Mucus and mast cells (arrows) were stained dark purple and light blue, respectively. Scale bars, 50 μm. **d** Whole lung lysates (30 μg of protein) from WT and *Adgrf5*^*−/−*^ mice (3, 5, 12, and 30 weeks of age) were analyzed by western blotting using antibodies specific for TPSAB1 (top) and α-tubulin (bottom). The band intensity was normalized to that of α-tubulin and is expressed as a value relative to that of corresponding WT mice. Values are presented as the mean ± SEM (*n* = 3). *p < 0.05 and ***p < 0.001. **e**–**g** Wright-Giemsa staining was performed on BAL cells from WT and *Adgrf5*^*−/−*^ mice (4 and 30 weeks of age). The numbers of macrophages (Mac), neutrophils (Neu), lymphocytes (Lym), and eosinophils (Eos) were counted. Results are expressed as the percentage of each inflammatory cell relative to total BAL cells, and are presented as the mean ± SEM (*n* = 3). *p < 0.05 and **p < 0.001. Representative images of BAL cells from 4-week-old WT and *Adgrf5*^*−/−*^ mice are shown in (**f**) and (**g**), respectively. The arrow indicates neutrophils. Scale bars, 50 μm. **h** Lung sections from *Adgrf5*^*−/−*^ mice at 10 weeks of age were stained with an Eosinophil-Mast Cell Stain Kit. Nuclei (blue purple) were counter-stained with hematoxylin. A representative image of an eosinophil (arrow) is shown. Scale bar, 50 μm

IL-4 and IL-13 are known to induce IgE synthesis by B cells and subsequent allergic responses [17]. Expectedly, serum IgE levels in *Adgrf5*^*−/−*^ mice were highly increased compared to those in WT mice at 30 weeks of age (19-fold, *p* < 0.001), whereas no such increase was detected at 3 and 8 weeks of age (Fig. 7a). Moreover, an accumulation of mast cells was observed in the peribronchiolar area of *Adgrf5*^*−/−*^ lungs at 30 weeks of age (Fig. 7c, arrows, compared to Fig. 7b). Western blot analysis of whole lung lysates showed that the levels of TPSAB1 (mast cell tryptase), a specific marker of mast cells, were elevated in *Adgrf5*^*−/−*^ lungs at 12 and 30 weeks of age (Fig. 7d). The increased levels of IL-4/IL-13 and serum IgE as well as mast cell accumulation, led us to predict the occurrence of eosinophilia. However, Giemsa staining of BAL cells showed that there was no increase in eosinophil numbers in *Adgrf5*^*−/−*^ lungs at 4 and 30 weeks of age (Fig. 7e). Rather, an accumulation of neutrophils and lymphocytes was evident in the BAL fluid of *Adgrf5*^*−/−*^ mice (Fig. 7e–g). Careful observation of lung sections stained with Astra blue and Vital new red revealed that only a few eosinophils infiltrated in the alveolar wall of *Adgrf5*^*−/−*^, but not WT, lungs at 10 (Fig. 7h) and 30 weeks of age (data not shown). These results suggest that loss of ADGRF5 leads to increased IgE production and the accumulation of mast cells and neutrophils in the bronchiolar interstitium and alveolar space, respectively.

### Increased expression of CCL2, S100A8, and S100A9 in the ECs of *Adgrf5*^−/−^ mice

**Fig. 8:**
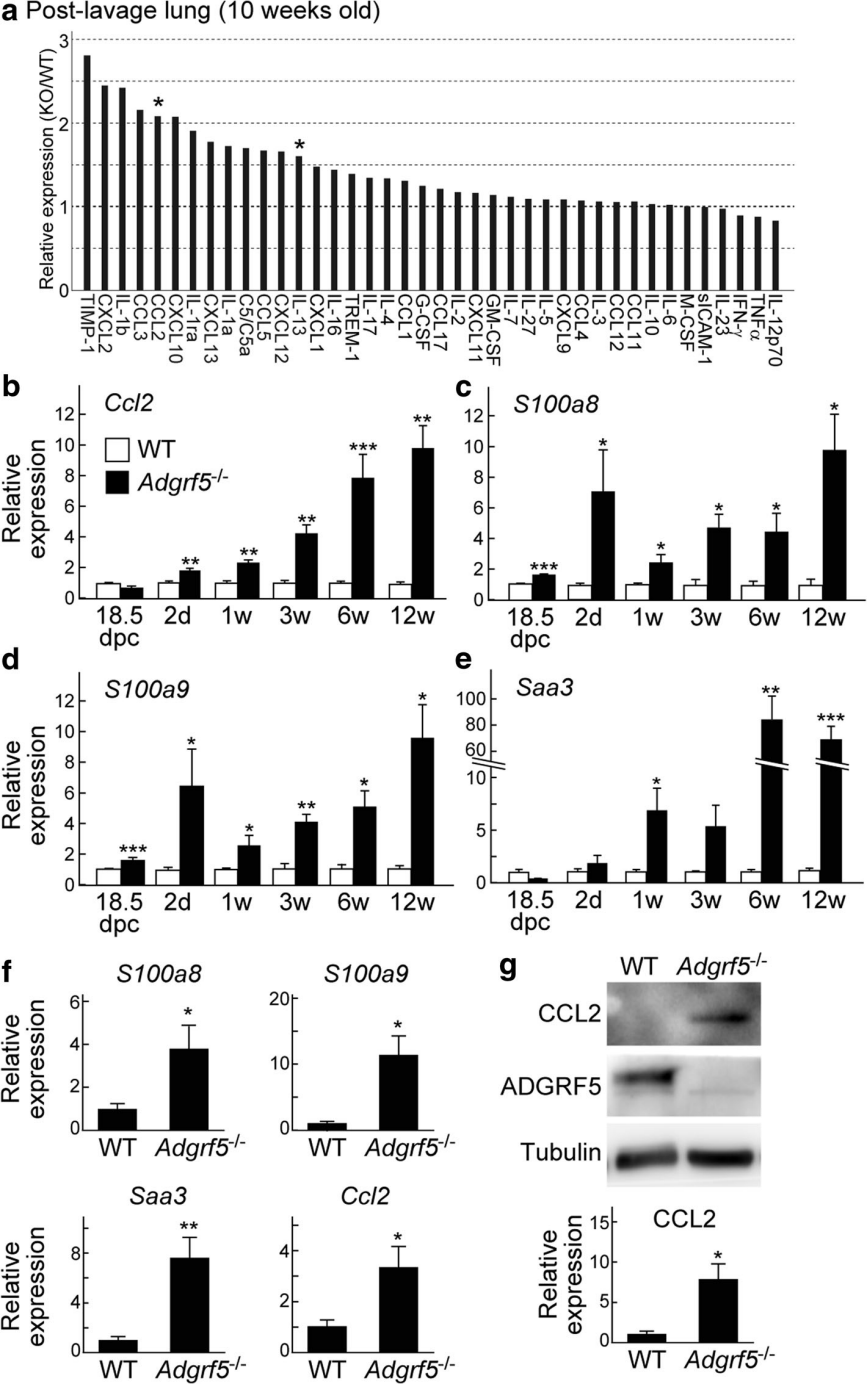
*Ccl2*, *S100a8*, *S100a9*, and *Saa3* expression is increased in *Adgrf5*^*−/−*^ lung ECs. **a** Cytokine and chemokine expression profile in lavage lungs from three mice (12-weeks-old), analyzed using a Proteome Profiler Mouse Cytokine Arrays as described in the Methods. The data from *Adgrf5*^*−/−*^ mice are expressed as values relative to those from WT mice. Values are presented as the average of duplicate measurements. Asterisks indicate CCL2 and IL-13. **b**–**e** The mRNA expression of *Ccl2* (**b**), *S100a8* (**c**), *S100a9* (**d**), and *Saa3* (**e**) was analyzed by qPCR in whole (18.5 days post-coitum and 2 days and 1 week of age) or post-lavage (3, 6, and 30 weeks of age) lungs from WT and *Adgrf5*^*−/−*^ mice. The data were normalized to *Gapdh* levels and are expressed as values relative to those from corresponding WT mice. Values are presented as the mean ± SEM (*n* = 3–6). *p < 0.05; **p < 0.01; ***p < 0.001. **f** The mRNA expression of *S100a8*, *S100a9*, *Saa3*, and *Ccl2* was analyzed by qPCR in primary lung ECs from WT and *Adgrf5*^*−/−*^ mice (1-week-old). The data were normalized to *Gapdh* levels and are expressed as values relative to those from WT mice. Values are presented as the mean ± SEM (*n* = 4–5). *p < 0.05 and **p < 0.005. **g** Whole cell lysates (10 μg of protein) from lung ECs were analyzed by western blotting using antibodies specific for CCL2 (top), ADGRF5 (middle), and α-tubulin (bottom). The band intensity of CCL2 was normalized to that of α-tubulin and expressed as a value relative to that from WT mice. Values are presented as mean ± SEM (*n* = 5). *p < 0.05

In a previous study, we showed that high levels of CCL2 are secreted from alveolar macrophages and are present in the BAL fluid of adult *Adgrf5*^*−/−*^ mice [12]. To analyze the cytokine and chemokine profile of lung tissues from both groups at 10 weeks of age, the lysates of post-lavage lungs were incubated with an antibody array that can detect 40 different cytokines/chemokines. The signal intensity of each antibody spot was measured and the *Adgrf5*^*−/−*^-to-WT signal ratio was calculated. In this experiment, a ratio of > 1.5 was considered increased production. As shown in Fig. 8a, a remarkable increase was detected for CCL2 and 13 other proteins including IL-1α and IL-1β, as well as their receptor antagonist (IL-1ra), IL-13, inflammatory chemokines (CCL3, CCL5, CXCL2, CXCL10, CXCL12, and CXCL13), tissue inhibitor of metalloprotease 1 (TIMP-1), and complement component 5 (C5/C5a).

Increased *Ccl2* expression was confirmed by qPCR, which indicated a 9.6-fold increase in expression (*p* < 0.01) in post-lavage lungs from *Adgrf5*^*−/−*^ mice compared to that in WT lungs at 12 weeks of age (Fig. 8b). In a previous study, increased CCL2 protein levels were detected in embryonic lungs of *Adgrf5*^*−/−*^ mice [12]. However, *Ccl2* mRNA expression in *Adgrf5*^*−/−*^ lungs was comparable to that in WT lungs at 18.5 dpc, but was upregulated in *Adgrf5*^*−/−*^ lungs compared to expression in WT lungs at 2 days of age (1.8-fold, *p* < 0.01); further, this difference gradually increased with age (Fig. 8b). S100A8 and S100A9 have proinflammatory functions, and have been shown to induce CCL2 expression [44–48]. We found that *S100a8* and *S100a9* mRNA levels were significantly increased in *Adgrf5*^*−/−*^ lungs at 18.5 dpc (1.6- and 1.7-fold, respectively, *p* < 0.001), as well as thereafter (Fig. 8c,d). In addition, the expression of *Saa3*, a downstream target of S100A8 and S100A9, was elevated in *Adgrf5*^*−/−*^ lungs at 1 week of age (Fig. 8e). These results suggest that CCL2 expression is increased in response to S100A8 and S100A9 stimulation in the lungs of late embryonic and early neonatal *Adgrf5*^*−/−*^ mice. It has been shown that CCL2 is released from lung ECs in response to inflammatory stimuli [49, 50]. We therefore examined if CCL2 is upregulated in the lung ECs of neonatal (1-week-old) *Adgrf5*^*−/−*^ mice. CD31-positive ECs were isolated from WT and *Adgrf5*^*−/−*^lungs and cultured for 1 week. Significantly increased CCL2 mRNA and protein expression was observed in *Adgrf5*^*−/−*^ ECs compared to that in WT ECs (3.3-fold, *p* < 0.05 and 7.8-fold, *p* < 0.05, respectively; Fig. 8f, g). In addition, elevated levels of *S100a8*, *S100a9*, and *Saa3* were also detected in *Adgrf5*^*−/−*^ ECs (3.8-fold, *p* < 0.05, 11.3-fold, *p* < 0.05, and 7.5-fold, *p* < 0.005, respectively; Fig. 8f). These results suggest that the loss of ADGRF5 in lung ECs induces the expression of proinflammatory regulators, specifically CCL2, S100A8, and S100A9, thereby contributing to the progression of airway inflammatory responses.

### Suppressive effect of a CCR2 antagonist on the expression of *S100a8*, *S100a9*, *Slc26a4*, and *Il5* in *Adgrf5*^−/−^ lungs

**Fig. 9:**
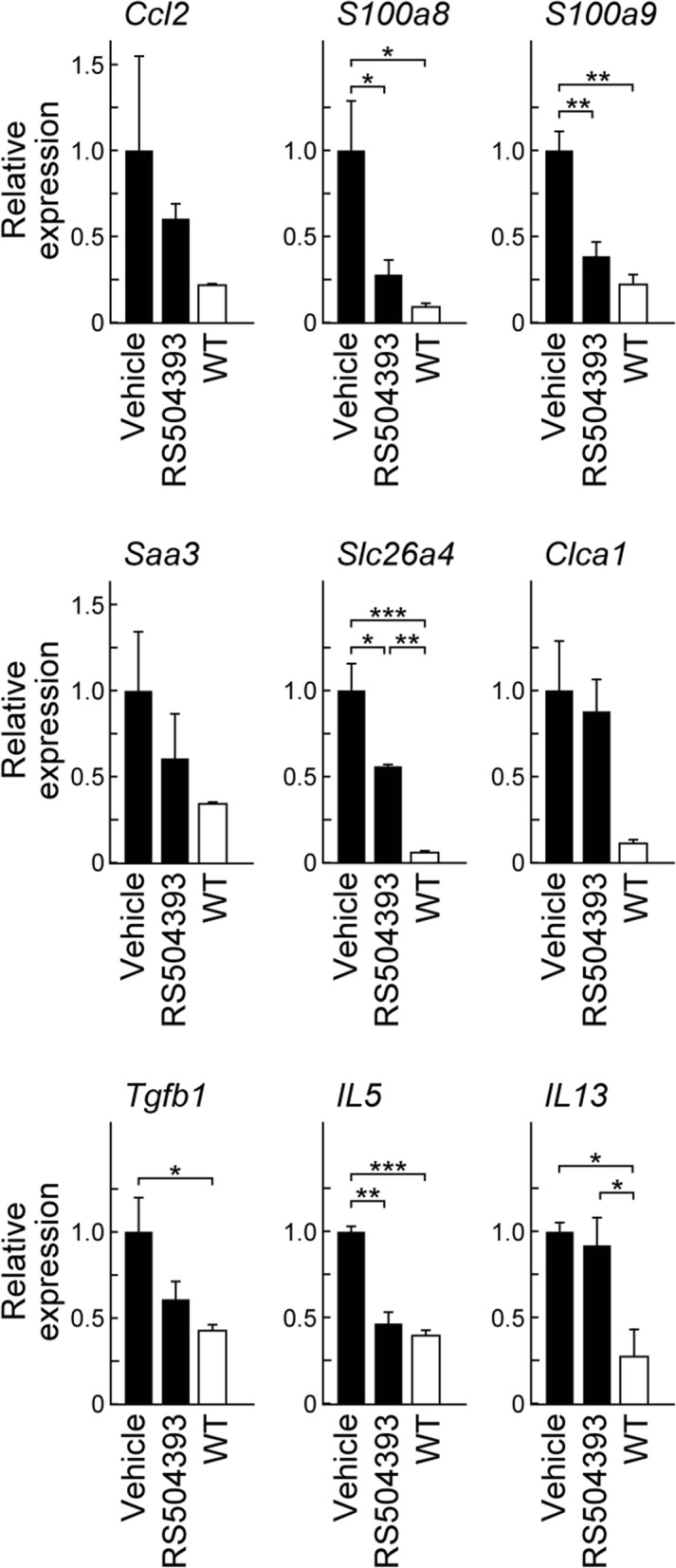
Suppressive effect of a CCR2 antagonist on the expression of *S100a8*, *S100a9*, *Slc26a4*, and *Il5* in *Adgrf5*^−/−^ lungs. Two-week-old *Adgrf5*^*−/−*^ mice were administered RS504393 (2 mg/kg body weight) or vehicle once daily via subcutaneous injection for 8 days. The mRNA expression of *Ccl2*, *S100a8*, *S100a9*, *Saa3*, *Slc26a4*, *Clca1*, *Tgfb1*, *Il5*, and *Il13 *was analyzed by qPCR using total RNA isolated from post-lavage lungs of injected mice and non-injected WT mice at 3 weeks of age. The data were normalized to *Gapdh* levels and are expressed as values relative to those from corresponding vehicle-treated *Adgrf5*^*−/−*^ mice. Values are presented as the mean ± SEM (*n* = 3–4). *p < 0.05; **p < 0.01; ***p < 0.001

To investigate if CCL2 has a role in the early events of airway inflammatory responses in *Adgrf5*^*−/−*^ lungs, we evaluated the effect of RS504393, a CCR2 antagonist, on the mRNA expression of genes upregulated in *Adgrf5*^*−/−*^ lungs at 3 weeks of age. Two-week-old *Adgrf5*^*−/−*^ mice were administered RS504393 (2 mg/kg body weight) or vehicle once daily via subcutaneous injection for 8 days. qPCR was performed using total RNA isolated from post-lavage lungs of injected mice and non-injected WT mice at 3 weeks of age. As shown in Fig. 9, RS504393 treatment strongly attenuated the expression of *S100a8*, *S100a9*, and *Il5* to WT levels and significantly decreased *Slc26a4* expression by 56%. The expression of *Saa3* and *Tgfb1* tended to decrease with RS504393 treatment, although this difference was not statistically significant. Further, there was no effect on the expression of *Ccl2*, *Clca1*, and *Il13*. These results suggest that CCL2–CCR2 signaling participates in the upregulation of the expression of *S100a8*, *S100a9*, *Il5*, and *Slc26a4* in *Adgrf5*^*−/−*^ lungs.

### No effect of CCL2 treatment on the expression of *MUC5AC*, *SLC26A4*, IL-25, or IL-33 in NCI-H292 cells

**Fig. 10:**
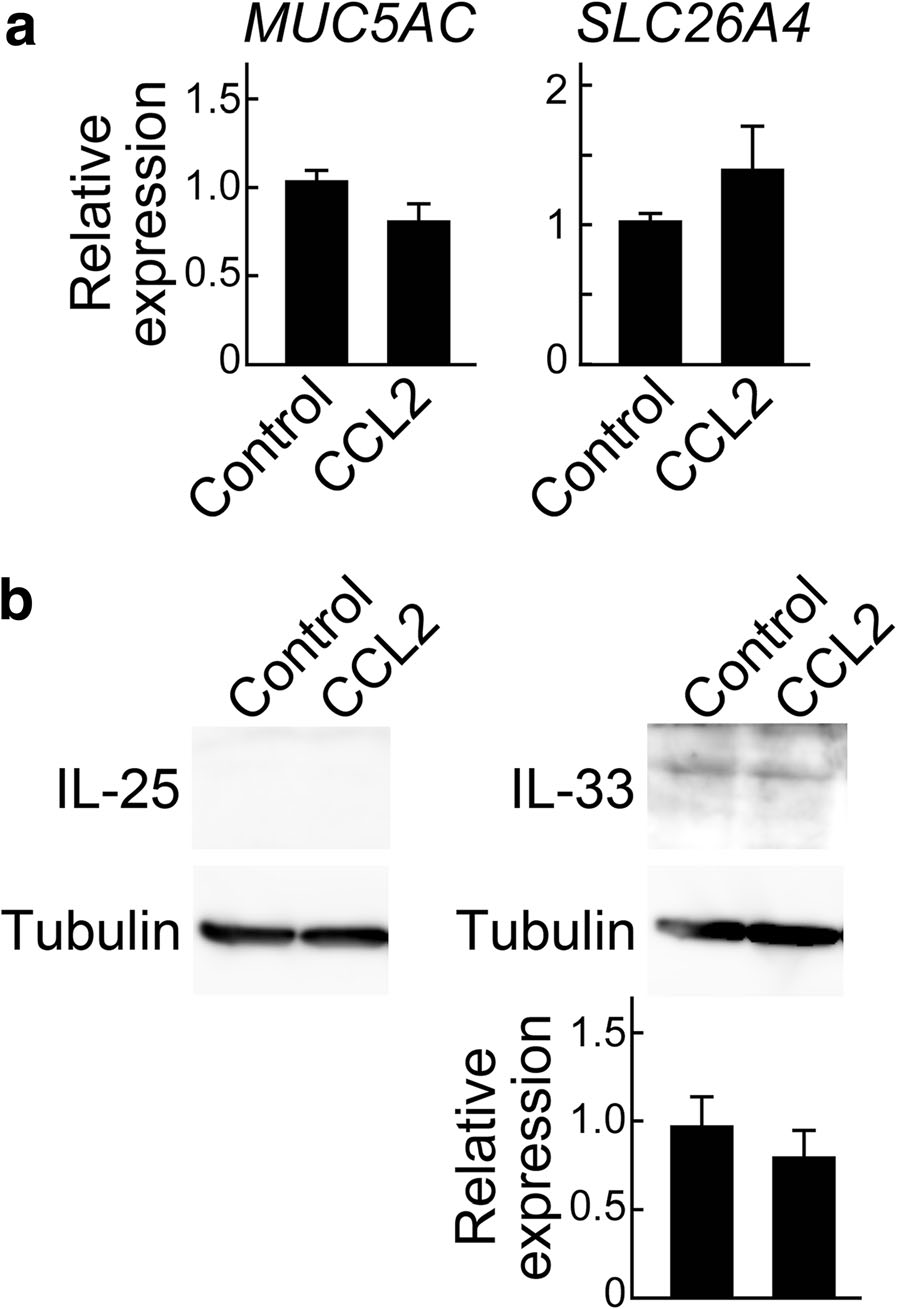
CCL2 treatment does not affect the expression of *MUC5AC*, *SLC26A4*, IL-25, or IL-33 in NCI-H292 cells. **a** The mRNA expression of *MUC5AC* and *SLC26A4* was analyzed by qPCR in NCI-H292 cells treated with or without 100 ng/ml CCL2 for 18 h. The data were normalized to *GAPDH* levels and are expressed as values relative to those from untreated control cells. Values are presented as the mean ± SEM (*n* = 3–5). **b** The protein expression of IL-25 and IL-33 was analyzed by western blotting in NCI-H292 cells treated with or without 100 ng/ml CCL2 for 18 h. Whole cell lysates (20 μg of protein) were analyzed by western blotting using antibodies specific for IL-25 (left top), IL-33 (right top), and α-tubulin (bottom). The expression of IL-25 was not detected. The band intensity of IL-33 was normalized to that of α-tubulin and expressed as a value relative to that from untreated control cells. Values are presented as mean ± SEM (*n* = 4)

To examine the possibility that CCL2 upregulates the genes related to mucus hypersecretion (*MUC5AC* and *SLC26A4*) and the epithelial cell-derived cytokines (IL-25 and IL-33) in airway epithelial cells, the human pulmonary mucoepidermoid carcinoma NCI-H292 cells were treated with or without 100 ng/ml CCL2 for 18 h. As shown in Fig. 10, there was no significant difference in the expression of *MUC5AC*, *SLC26A4*, IL-25, or IL-33 between untreated and CCL2-treated cells.

## Discussion

This study provides evidence suggesting that ADGRF5 ablation in mice leads to the development of airway inflammation in lungs. Histochemical studies clearly showed an increase in mucus production and the appearance of mucus-producing cells in the bronchiolar epithelium of *Adgrf5*^*−/−*^ mice. Excessive mucus accumulation in the airway lumens is a common feature of asthma and COPD and is caused by an increase in the number of mucus-secreting goblet cells [30]. In mice, intralobular bronchioles are predominantly lined with two types of epithelial cells, namely ciliated cells and non-ciliated secretory club (Clara) cells, whereas goblet cells are sparse. Goblet cells are known to be transdifferentiated primarily from club cells [34, 51]. An increase in the production and secretion of MUC5AC by goblet cells mainly contributes to airway mucus accumulation [52, 53]. Therefore, the increased number of Alcian blue- and MUC5AC-positive cells in *Adgrf5*^*−/−*^ bronchioles indicates the occurrence of mucous (goblet) cell metaplasia. This interpretation is supported by the finding of high SPDEF and low FOXA2 expression in the *Adgrf5*^*−/−*^ bronchiolar epithelium. Another supportive finding was the elevated expression of *Clca1*, which encodes a goblet cell-specific protein. CLCA1 is a secreted protein that signals in an autocrine and paracrine fashion to induce mucus hyperproduction. Extracellular CLCA1 activates the calcium-dependent chloride channel TMEM16A, and the resulting chloride currents are likely to induce MUC5AC expression through the activation of mitogen activated protein kinase 13 (MAPK13) signaling [54–56]. Furthermore, the respiratory acidosis observed in *Adgrf5*^*−/−*^ mice suggests that mucus hyperproduction likely causes airway obstruction and consequent airflow limitations as seen in asthma and COPD. However, we cannot exclude the possibility that abnormal pulmonary surfactant accumulation and alveolar obstruction might cause ineffective gas exchange.

High expression of type 2 cytokines (*Il4*, *Il5*, and *Il13*), IL-25, and IL-33 was detected in the lungs of *Adgrf5*^*−/−*^ mice. Among them, IL-4, IL-5, and IL-13 play key roles in immune responses during the pathogenesis of allergic inflammation. *Adgrf5*^*−/−*^ mice exhibited elevated serum IgE levels and mast cell infiltration into the bronchiolar interstitium, which was likely induced by IL-4 and/or IL-13. Importantly, IL-13 is essential for mucous cell metaplasia and mucus hypersecretion, and functions through direct effects on airway epithelial cells [40, 57–59]. The binding of IL-13 to its epithelial receptor triggers the activation of STAT6, which upregulates SPDEF and CLCA1 [60, 61]. Therefore, the finding that *Il13* mRNA and protein levels were increased along with elevated SPDEF and *Clca1* expression in *Adgrf5*^*−/−*^ lungs suggests that mucous cell metaplasia is mediated at least in part by the epithelial effects of IL-13. Recent studies reported that SLC26A4, an anion transporter present on the apical side of airway epithelial cells, is a downstream target of the IL-4/IL-13–STAT6 signaling pathway [41–43, 62]. SLC26A4 has been implicated in the pathogenesis of COPD and asthma, including mucus hypersecretion, airway allergic inflammation, and airway hyperreactivity [42]. The transepithelial transport of thiocyanate (SCN^−^), via SLC26A4, leads to the production of hypothiocyanite (OSCN^−^) in the airway lumen, which contributes to the innate host defense at low doses or to epithelial damage and type 2 inflammation at high doses [63]. The elevated expression of *Slc26a4* in *Adgrf5*^*−/−*^ lungs thus reflects IL-4/IL-13-mediated mucus hypersecretion and allergic inflammation. IL-5 is a potent inducer of eosinophil recruitment and activation during airway allergic inflammation [18]. However, eosinophilia was not detected in *Adgrf5*^*−/−*^ lungs even though only a small number of eosinophils were recruited to the alveolar wall. Rather, a significant accumulation of neutrophils was observed, which is consistent with the previous report by Bridges et al. [9]. Since neutrophilic inflammation is common during COPD, severe asthma, cystic fibrosis, and bronchiectasis, all of which involve airway mucus hypersecretion [64], infiltrating neutrophils, in addition to eosinophils, might contribute to the progression of airway inflammation in *Adgrf5*^*−/−*^ mice.

Increased type 2 cytokine production has long been attributed to T_H_2 cells. However, increasing evidence indicates that ILC2s are the primary sources of IL-4, IL-5, and IL-13, and that these cells can induce a type 2 immune response, which is accompanied by airway mucus hypersecretion [19, 20]. ILC2s are potently activated by epithelial cell-derived IL-25 and IL-33, both of which are released in response to allergens, viral infection, and epithelial injury [21, 22, 65]. Our results thus suggest that the increased release of IL-25 and IL-33 likely enhances type 2 inflammation in *Adgrf5*^*−/−*^ lungs, at as early as 12 weeks of age, thereby leading to mucus hyperproduction. However, it is not clear how these two epithelial cytokines are upregulated and released. This is most likely to be an indirect effect of ADGRF5 disruption because *Adgrf5* mRNA expression was not detected in the bronchiolar epithelium. As discussed, SLC26A4 might be involved in IL-25 and IL-33 upregulation through tissue damage. A recent study showed that oxidative stress enhances IL-33 expression in primary human bronchial epithelial cells and NCI-H292 cells via MAPK and NF-κB signaling pathways [66]. We previously showed that the disruption of *Adgrf5* leads to the excessive production and release of reactive oxygen species by alveolar macrophages, exposing the lung parenchyma to oxidative stress [12]. An accumulation of alveolar macrophages was observed in the alveoli of *Adgrf5*^*−/−*^ mice as early as 2–3 weeks of age [10, 11]. Therefore, it is speculated that alveolar macrophage-mediated oxidative stress might directly induce IL-33 expression and/or cause epithelial damage leading to the release of IL-25 and IL-33.

*Adgrf5*^*−/−*^ mice were found to exhibit mild interstitial fibrosis around the bronchioles with increased expression of *Tgfb1*, *Col1a1*, *Fn1*, and *Tnc*. TGF-β has an important role in airway remodeling including subepithelial fibrosis, airway smooth muscle thickening, and mucous cell metaplasia [67, 68]. Subepithelial fibrosis is characterized by the extensive deposition of extracellular matrix (ECM) such as collagen I, collagen III, fibronectin, and tenascin-C [69, 70]. TGF-β also promotes the differentiation of fibroblasts into myofibroblasts and ECM protein production by these cells. This ligand is produced by many types of lung cells including epithelial cells, fibroblasts, eosinophils, and macrophages [71–74]. TGF-β is secreted as a functionally inactive precursor that is complexed with latent TGF-β-binding protein. Moreover, MMP-2 and MMP-9 are known to activate TGF-β by proteolytic cleavage [75]. We detected increased levels of the active form of TGF-β1 in *Adgrf5*^*−/−*^ lungs. In addition, we previously showed that MMP-2 and MMP-9 are highly expressed in and secreted from the alveolar macrophages of *Adgrf5*^*−/−*^ mice [12]. These findings suggest that these proteases might be largely involved in TGF-β1 activation. ECM turnover is tightly controlled by collagen-degrading MMPs and their endogenous inhibitors, TIMPs. An imbalance between these two opposing activities is thought to lead to fibrosis, tissue remodeling, and inflammation [76]. Thus, high levels of TIMP-1 in lavage lungs (Fig. 8a) and BAL fluid [12] might exaggerate subepithelial fibrosis in *Adgrf5*^*−/−*^ mice.

*Adgrf5*^*−/−*^ mice show several key features of pulmonary alveolar proteinosis (PAP), such as the accumulation of pulmonary surfactant and foamy alveolar macrophages in the alveolar space. However, mucus hyperproduction and neutrophilic inflammation are not generally observed in PAP patients with GM-CSF autoantibodies or in mouse models lacking GM-CSF [64, 77]. This phenotype results from the loss of GM-CSF receptor signaling, which is required for alveolar macrophage and neutrophil differentiation and immune functions [78]. In contrast, GM-CSF signaling in alveolar macrophages is not affected in *Adgrf5*^*−/−*^ mice [8]. Therefore, alveolar macrophages from *Adgrf5*^*−/−*^ mice retain the ability to induce emphysema symptoms and airway inflammation. Mice lacking *Sftpc* (surfactant protein C) also exhibit the massive accumulation of pulmonary surfactant and foamy alveolar macrophages. *Sftpc*^*−/−*^ mice exhibit emphysematous destruction of alveolar walls, airway mucus hyperproduction, and increased MMP2/9 release without neutrophilia or cytokine upregulation (TNF-α, IL-1β, IL-13, and IL-6) [79], whereas increased expression of IL-1β and IL-13 was detected in *Adgrf5*^*−/−*^ lungs (Fig. 8a). Although the cause of the discrepancies between phenotypes of *Adgrf5*^*−/−*^ and *Sftpc*^−/−^ mice is unclear, airway inflammation induced by *Adgrf5*^*−/−*^ disruption is likely to be mediated by a mechanism independent of pulmonary surfactant and alveolar macrophages.

**Fig. 11:**
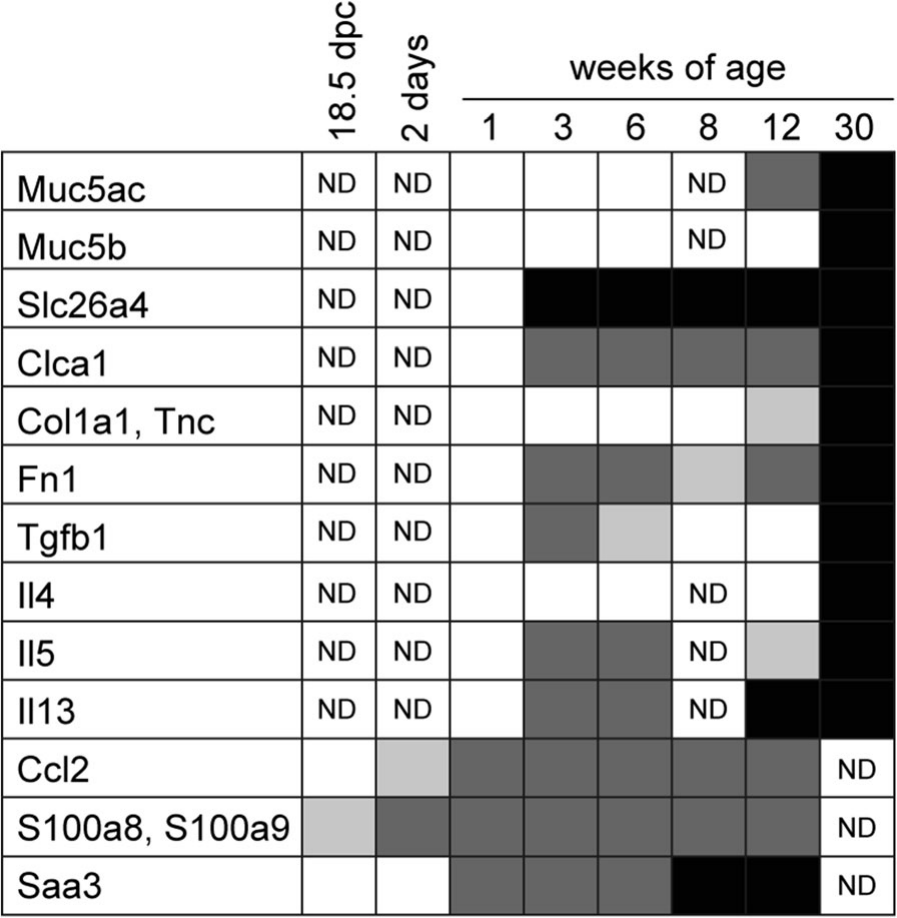
Summary of gene expression profiles comparing WT and *Adgrf5*^*−/−*^ lungs. Dark or light gray boxes represent genes exhibiting a greater than or less than two-fold increase in expression, respectively, in *Adgrf5*^*−/−*^ lungs at the indicated age. A black box represents a > 10-fold increase in gene expression of between WT and *Adgrf5*^*−/−*^ lungs. An open box represents no significant change in gene expression between WT and *Adgrf5*^*−/−*^ lungs. ND, not determined

Figure 11 summarizes the expression profiles of genes analyzed in this study. Based on our results, mucous cell metaplasia, mucus hyperproduction, and respiratory acidosis occur at as early as 10 weeks of age, which is followed by increased IgE production, mast cell accumulation, and subepithelial fibrosis by 30 weeks of age. It is noteworthy that upregulation of *Ccl2*, *S100a8*, and *S100a9* expression occurs during embryonic (18.5 dpc) and neonatal (2 days of age) stages. CCL2 is closely associated with the pathogenesis of airway inflammation [80, 81]. Accordingly, targeted disruption and antibody-mediated neutralization of CCL2 reduces airway allergic inflammation and airway hyperreactivity in allergen-challenged mice [82–84]. S100A8 and S100A9 form a heterodimer (S100A8/A9 or calprotectin) and act as a proinflammatory mediator that is associated with acute and chronic inflammation, cancer cell metastasis, and tumorigenesis [85, 86]. In particular, S100A8 and S100A9 are involved in MUC5AC upregulation in human airway epithelial cells [87]. Taken together, these results suggest that CCL2, S100A8, and S100A9 might be involved in the immediate early phase of airway inflammation pathogenesis. Interestingly, RS504393 treatment suppressed the upregulation of *S100a8*, *S100a9*, *Slc26a4*, and *Il5* in *Adgrf5*^*−/−*^ lungs at 3 weeks of age. This finding suggests that CCL2–CCR2 signaling might function in gene regulation associated with mucus hyperproduction and type 2 inflammation (Additional file 1: Figure S1). However, CCL2 did not directly upregulate the expression of *MUC5AC*, *SLC26A4*, IL-25, and IL-33 in NCI-H292 cells under our experimental conditions. The underlying mechanism should be addressed in a future study.

It is likely that alveolar macrophages are not the source of CCL2 in animals younger than 2–3 weeks of age, because their accumulation cannot be detected in embryonic and neonatal *Adgrf5*^*−/−*^ mice [10, 11]. Interestingly, we found that CCL2 mRNA and protein expression was upregulated in primary lung ECs prepared from 1-week-old *Adgrf5*^*−/−*^ mice. Furthermore, the expression of *S100a8* and *S100a9* was also increased in these ECs, as well as in the lungs of *Adgrf5*^*−/−*^ embryos. S100A8/A9 or S100A8 has been shown to induce CCL2 production in ECs [44], fibrocytes [47], and alveolar epithelial cells [88]. In addition, CCL2, secreted by primary tumor cells, stimulates lung ECs to release S100A8 and S100A9, leading to paracrine upregulation of SAA3, and consequently the formation of the premetastatic niche [89, 90]. CCL2 and S100A8/A9 might therefore form a positive feedback loop in lung ECs. To support this hypothesis, RS504393 treatment abrogated the upregulation of *S100a8* and *S100a9* in *Adgrf5*^*−/−*^ lungs. Since ECs are sites of ADGRF5 expression in a variety of tissues [10, 11], the lack of endothelial ADGRF5 might disrupt intracellular signaling pathways that regulate CCL2, S100A8, and S100A9 expression, thereby stimulating a positive feedback loop. However, we cannot rule out the possible contribution of AT2 epithelial cells that strongly express ADGRF5 in a similar manner. It has been shown that the lung endothelium does not contribute to pulmonary surfactant homeostasis, and EC-specific *Adgrf5* knockout does not result in the accumulation of alveolar macrophages [6, 11]. In addition, AT2 cells represent a significant source of IL-33, and a lack of G_q/11_ signaling promotes IL-33-mediated alveolar macrophage activation and emphysema [91, 92]. Therefore, CCL2 expression and the subsequent development of airway inflammation might result from the synergistic effects of ECs, AT2 epithelial cells, and alveolar macrophages.

## Conclusions

Our study showed for the first time that *Adgrf5*^*−/−*^ mice exhibit characteristic features of airway inflammation, including mucous cell metaplasia, mucus hyperproduction, type 2 inflammation, subepithelial fibrosis, and respiratory acidosis. Expression profiles of genes/proteins involved in airway inflammation suggested that these inflammatory phenotypes are mediated by the secondary effects of *Adgrf5* deletion, and that type 2 cytokines are likely to play an important role in pathogenesis. In addition, proinflammatory CCL2, S100A8, and S100A9, released at least in part by ECs, might be involved in the onset and progression of these airway inflammatory phenotypes. These results and this model could be helpful for studying the pathogenesis of chronic airway inflammation, such as COPD and asthma, and suggest that ADGRF5 is a potential biomarker and therapeutic target. Further studies are needed to clarify the underlying mechanism and to better understand the role of ADGRF5 in EC functions, as well as in immune regulation in the lung.

## Additional file

Additional file 1: **Figure S1.** Schematic diagram of a possible mechanism leading to the airway phenotypes of *Adgrf5*^*−/−*^ mice. *Adgrf5* deletion increases CCL2 expression in the lung endothelium during embryonic and neonatal stages. CCL2 is involved in the upregulation of *Slc26a4*, *Il5*, and possibly *Tgfb1*, thereby contributing to the onset and/or progression of mucus hypersecretion, type 2 inflammation, and fibrosis, respectively (red arrows). *Adgrf5* deletion also causes abnormal surfactant homeostasis, which leads to recruitment and activation of alveolar macrophages. MMPs and reactive oxygen species (ROS) released from alveolar macrophages might induce the release of IL-25 and IL-33, and subepithelial fibrosis. IL-25 and IL-33 are likely to increase the production of type 2 cytokines (IL-4, IL-5, and IL-13), which promotes mucous cell metaplasia, mucus hypersecretion, IgE production, mast cell accumulation, and fibrosis. (TIF 208 kb)

## Abbreviations

ADGRF5: Adhesion G protein-coupled receptor F5
AT2: Alveolar type II
BAL: Bronchoalveolar lavage
BSA: Bovine serum albumin
CCL: C-C motif chemokine ligand
CCR2: C-C chemokine receptor type 2
COPD: Chronic obstructive pulmonary disease
dpc: Day(s) post-coitum
EC: Endothelial cell
ECM: Extracellular matrix
FOX2A: Forkhead box A2
GM-CSF: Granulocyte macrophage colony-stimulating factor
GPCR: G protein-coupled receptor
Ig: Immunoglobulin
IL: Interleukin
ILC2: Type 2 innate lymphoid cell
MMP: Matrix metalloprotease
PAP: Pulmonary alveolar proteinosis
PBS: Phosphate-buffered saline
qPCR: Quantitative real-time PCR
SPDEF: SAM-pointed domain-containing Ets-like factor
TGF: Transforming growth factor
T_H_2: T helper 2
TIMP: Tissue inhibitor of metalloprotease
WT: Wildtype

## References

[1] Hamann J, Aust G, Arac D, Engel FB, Formstone C, Fredriksson R, Hall RA, Harty BL, Kirchhoff C, Knapp B (2015). International Union of Basic and Clinical Pharmacology. XCIV. Adhesion G protein-coupled receptors. Pharmacol Rev.

[2] Abe J, Suzuki H, Notoya M, Yamamoto T, Hirose S (1999). Ig-hepta, a novel member of the G protein-coupled hepta-helical receptor (GPCR) family that has immunoglobulin-like repeats in a long N-terminal extracellular domain and defines a new subfamily of GPCRs. J Biol Chem.

[3] Fukuzawa T, Hirose S (2006). Multiple processing of Ig-Hepta/GPR116, a G protein-coupled receptor with immunoglobulin (Ig)-like repeats, and generation of EGF2-like fragment. J Biochem.

[4] Tang X, Jin R, Qu G, Wang X, Li Z, Yuan Z, Zhao C, Siwko S, Shi T, Wang P (2013). GPR116, an adhesion G-protein-coupled receptor, promotes breast cancer metastasis via the Gα_q_-p63RhoGEF-Rho GTPase pathway. Cancer Res.

[5] Demberg LM, Winkler J, Wilde C, Simon KU, Schon J, Rothemund S, Schoneberg T, Promel S, Liebscher I (2017). Activation of adhesion G protein-coupled receptors: agonist specificity of stachel sequence-derived peptides. J Biol Chem.

[6] Brown K, Filuta A, Ludwig MG, Seuwen K, Jaros J, Vidal S, Arora K, Naren AP, Kandasamy K, Parthasarathi K (2017). Epithelial Gpr116 regulates pulmonary alveolar homeostasis via G_q/11_ signaling. JCI Insight.

[7] Nie T, Hui X, Gao X, Li K, Lin W, Xiang X, Ding M, Kuang Y, Xu A, Fei J (2012). Adipose tissue deletion of Gpr116 impairs insulin sensitivity through modulation of adipose function. FEBS Lett.

[8] Yang MY, Hilton MB, Seaman S, Haines DC, Nagashima K, Burks CM, Tessarollo L, Ivanova PT, Brown HA, Umstead TM (2013). Essential regulation of lung surfactant homeostasis by the orphan G protein-coupled receptor GPR116. Cell Rep.

[9] Bridges JP, Ludwig MG, Mueller M, Kinzel B, Sato A, Xu Y, Whitsett JA, Ikegami M (2013). Orphan G protein-coupled receptor GPR116 regulates pulmonary surfactant pool size. Am J Respir Cell Mol Biol.

[10] Fukuzawa T, Ishida J, Kato A, Ichinose T, Ariestanti DM, Takahashi T, Ito K, Abe J, Suzuki T, Wakana S (2013). Lung surfactant levels are regulated by Ig-Hepta/GPR116 by monitoring surfactant protein D. PLoS One.

[11] Niaudet C, Hofmann JJ, Mae MA, Jung B, Gaengel K, Vanlandewijck M, Ekvarn E, Salvado MD, Mehlem A, Al Sayegh S (2015). Gpr116 receptor regulates distinctive functions in pneumocytes and vascular endothelium. PLoS One.

[12] Ariestanti DM, Ando H, Hirose S, Nakamura N (2015). Targeted disruption of Ig-Hepta/Gpr116 causes emphysema-like symptoms that are associated with alveolar macrophage activation. J Biol Chem.

[13] Tzortzaki EG, Proklou A, Siafakas NM (2011). Asthma in the elderly: can we distinguish it from COPD?. J Allergy (Cairo).

[14] Saha S, Brightling CE (2006). Eosinophilic airway inflammation in COPD. Int J Chron Obstruct Pulmon Dis.

[15] Thomson NC (2016). Novel approaches to the management of noneosinophilic asthma. Ther Adv Respir Dis.

[16] Fahy JV (2015). Type 2 inflammation in asthma--present in most, absent in many. Nat Rev Immunol.

[17] Oettgen HC (2000). Regulation of the IgE isotype switch: new insights on cytokine signals and the functions of epsilon germline transcripts. Curr Opin Immunol.

[18] Takatsu K, Nakajima H (2008). IL-5 and eosinophilia. Curr Opin Immunol.

[19] Kabata H, Moro K, Koyasu S, Asano K (2015). Group 2 innate lymphoid cells and asthma. Allergol Int.

[20] Cavagnero K, Doherty TA (2017). Cytokine and lipid mediator regulation of group 2 innate lymphoid cells (ILC2s) in human allergic airway disease. J Cytokine Biol.

[21] Neill DR, Wong SH, Bellosi A, Flynn RJ, Daly M, Langford TK, Bucks C, Kane CM, Fallon PG, Pannell R (2010). Nuocytes represent a new innate effector leukocyte that mediates type-2 immunity. Nature.

[22] Moro K, Yamada T, Tanabe M, Takeuchi T, Ikawa T, Kawamoto H, Furusawa J, Ohtani M, Fujii H, Koyasu S (2010). Innate production of T_H_2 cytokines by adipose tissue-associated c-Kit^+^Sca-1^+^ lymphoid cells. Nature.

[23] Barnes PJ (2016). Inflammatory mechanisms in patients with chronic obstructive pulmonary disease. J Allergy Clin Immunol.

[24] Nadel JA (2000). Role of neutrophil elastase in hypersecretion during COPD exacerbations, and proposed therapies. Chest.

[25] Fricker M, Deane A, Hansbro PM (2014). Animal models of chronic obstructive pulmonary disease. Expert Opin Drug Discov.

[26] Nials AT, Uddin S (2008). Mouse models of allergic asthma: acute and chronic allergen challenge. Dis Model Mech.

[27] Saito K, Nakamura N, Ito Y, Hoshijima K, Esaki M, Zhao B, Hirose S (2010). Identification of zebrafish Fxyd11a protein that is highly expressed in ion-transporting epithelium of the gill and skin and its possible role in ion homeostasis. Front Physiol.

[28] Aihara T, Nakamura N, Honda S, Hirose S (2009). A novel potential role for gametogenetin-binding protein 1 (GGNBP1) in mitochondrial morphogenesis during spermatogenesis in mice. Biol Reprod.

[29] Bonser LR, Erle DJ (2017). Airway mucus and asthma: the role of MUC5AC and MUC5B. J Clin Med.

[30] Curran DR, Cohn L (2010). Advances in mucous cell metaplasia: a plug for mucus as a therapeutic focus in chronic airway disease. Am J Respir Cell Mol Biol.

[31] Hoshino M, Morita S, Iwashita H, Sagiya Y, Nagi T, Nakanishi A, Ashida Y, Nishimura O, Fujisawa Y, Fujino M (2002). Increased expression of the human Ca^2+^-activated cl^−^ channel 1 (CaCC1) gene in the asthmatic airway. Am J Respir Crit Care Med.

[32] Gibson A, Lewis AP, Affleck K, Aitken AJ, Meldrum E, Thompson N (2005). hCLCA1 and mCLCA3 are secreted non-integral membrane proteins and therefore are not ion channels. J Biol Chem.

[33] Sala-Rabanal M, Yurtsever Z, Berry KN, Brett TJ (2015). Novel roles for chloride channels, exchangers, and regulators in chronic inflammatory airway diseases. Mediat Inflamm.

[34] Chen G, Korfhagen TR, Xu Y, Kitzmiller J, Wert SE, Maeda Y, Gregorieff A, Clevers H, Whitsett JA (2009). SPDEF is required for mouse pulmonary goblet cell differentiation and regulates a network of genes associated with mucus production. J Clin Invest.

[35] Wan H, Kaestner KH, Ang SL, Ikegami M, Finkelman FD, Stahlman MT, Fulkerson PC, Rothenberg ME, Whitsett JA (2004). Foxa2 regulates alveolarization and goblet cell hyperplasia. Development.

[36] Chen G, Wan H, Luo F, Zhang L, Xu Y, Lewkowich I, Wills-Karp M, Whitsett JA (2010). Foxa2 programs Th2 cell-mediated innate immunity in the developing lung. J Immunol.

[37] Epstein SK, Singh N (2001). Respiratory acidosis. Respir Care.

[38] Temann UA, Prasad B, Gallup MW, Basbaum C, Ho SB, Flavell RA, Rankin JA (1997). A novel role for murine IL-4 in vivo: induction of MUC5AC gene expression and mucin hypersecretion. Am J Respir Cell Mol Biol.

[39] Zhou Y, Dong Q, Louahed J, Dragwa C, Savio D, Huang M, Weiss C, Tomer Y, McLane MP, Nicolaides NC, Levitt RC (2001). Characterization of a calcium-activated chloride channel as a shared target of Th2 cytokine pathways and its potential involvement in asthma. Am J Respir Cell Mol Biol.

[40] Kuperman DA, Huang X, Koth LL, Chang GH, Dolganov GM, Zhu Z, Elias JA, Sheppard D, Erle DJ (2002). Direct effects of interleukin-13 on epithelial cells cause airway hyperreactivity and mucus overproduction in asthma. Nat Med.

[41] Pedemonte N, Caci E, Sondo E, Caputo A, Rhoden K, Pfeffer U, Di Candia M, Bandettini R, Ravazzolo R, Zegarra-Moran O, Galietta LJ (2007). Thiocyanate transport in resting and IL-4-stimulated human bronchial epithelial cells: role of pendrin and anion channels. J Immunol.

[42] Nakao I, Kanaji S, Ohta S, Matsushita H, Arima K, Yuyama N, Yamaya M, Nakayama K, Kubo H, Watanabe M (2008). Identification of pendrin as a common mediator for mucus production in bronchial asthma and chronic obstructive pulmonary disease. J Immunol.

[43] Nofziger C, Vezzoli V, Dossena S, Schonherr T, Studnicka J, Nofziger J, Vanoni S, Stephan S, Silva ME, Meyer G, Paulmichl M (2011). STAT6 links IL-4/IL-13 stimulation with pendrin expression in asthma and chronic obstructive pulmonary disease. Clin Pharmacol Ther.

[44] Ehlermann P, Eggers K, Bierhaus A, Most P, Weichenhan D, Greten J, Nawroth PP, Katus HA, Remppis A (2006). Increased proinflammatory endothelial response to S100A8/A9 after preactivation through advanced glycation end products. Cardiovasc Diabetol.

[45] Deguchi A, Tomita T, Ohto U, Takemura K, Kitao A, Akashi-Takamura S, Miyake K, Maru Y (2016). Eritoran inhibits S100A8-mediated TLR4/MD-2 activation and tumor growth by changing the immune microenvironment. Oncogene.

[46] Miller RE, Belmadani A, Ishihara S, Tran PB, Ren D, Miller RJ, Malfait AM (2015). Damage-associated molecular patterns generated in osteoarthritis directly excite murine nociceptive neurons through toll-like receptor 4. Arthritis Rheumatol.

[47] Nishikawa Y, Kajiura Y, Lew JH, Kido JI, Nagata T, Naruishi K (2017). Calprotectin induces IL-6 and MCP-1 production via toll-like receptor 4 signaling in human gingival fibroblasts. J Cell Physiol.

[48] Schenten V, Plancon S, Jung N, Hann J, Bueb JL, Brechard S, Tschirhart EJ, Tolle F (2018). Secretion of the phosphorylated form of S100A9 from neutrophils is essential for the Proinflammatory functions of extracellular S100A8/A9. Front Immunol.

[49] Miller JD, Benjamin JT, Kelly DR, Frank DB, Prince LS (2010). Chorioamnionitis stimulates angiogenesis in saccular stage fetal lungs via CC chemokines. Am J Physiol Lung Cell Mol Physiol.

[50] Farahi N, Paige E, Balla J, Prudence E, Ferreira RC, Southwood M, Appleby SL, Bakke P, Gulsvik A, Litonjua AA (2017). Neutrophil-mediated IL-6 receptor trans-signaling and the risk of chronic obstructive pulmonary disease and asthma. Hum Mol Genet.

[51] Pardo-Saganta A, Law BM, Gonzalez-Celeiro M, Vinarsky V, Rajagopal J (2013). Ciliated cells of pseudostratified airway epithelium do not become mucous cells after ovalbumin challenge. Am J Respir Cell Mol Biol.

[52] Zuhdi Alimam M, Piazza FM, Selby DM, Letwin N, Huang L, Rose MC (2000). Muc-5/5ac mucin messenger RNA and protein expression is a marker of goblet cell metaplasia in murine airways. Am J Respir Cell Mol Biol.

[53] Young HW, Williams OW, Chandra D, Bellinghausen LK, Perez G, Suarez A, Tuvim MJ, Roy MG, Alexander SN, Moghaddam SJ (2007). Central role of Muc5ac expression in mucous metaplasia and its regulation by conserved 5′ elements. Am J Respir Cell Mol Biol.

[54] Alevy YG, Patel AC, Romero AG, Patel DA, Tucker J, Roswit WT, Miller CA, Heier RF, Byers DE, Brett TJ, Holtzman MJ (2012). IL-13-induced airway mucus production is attenuated by MAPK13 inhibition. J Clin Invest.

[55] Lin J, Jiang Y, Li L, Liu Y, Tang H, Jiang D (2015). TMEM16A mediates the hypersecretion of mucus induced by Interleukin-13. Exp Cell Res.

[56] Sala-Rabanal M, Yurtsever Z, Nichols CG, Brett TJ (2015). Secreted CLCA1 modulates TMEM16A to activate Ca^2+^-dependent chloride currents in human cells. elife.

[57] Laoukili J, Perret E, Willems T, Minty A, Parthoens E, Houcine O, Coste A, Jorissen M, Marano F, Caput D, Tournier F (2001). IL-13 alters mucociliary differentiation and ciliary beating of human respiratory epithelial cells. J Clin Invest.

[58] Kuperman DA, Huang X, Nguyenvu L, Holscher C, Brombacher F, Erle DJ (2005). IL-4 receptor signaling in Clara cells is required for allergen-induced mucus production. J Immunol.

[59] Zhen G, Park SW, Nguyenvu LT, Rodriguez MW, Barbeau R, Paquet AC, Erle DJ (2007). IL-13 and epidermal growth factor receptor have critical but distinct roles in epithelial cell mucin production. Am J Respir Cell Mol Biol.

[60] Thai P, Chen Y, Dolganov G, Wu R (2005). Differential regulation of MUC5AC/Muc5ac and hCLCA-1/mGob-5 expression in airway epithelium. Am J Respir Cell Mol Biol.

[61] Yu H, Li Q, Kolosov VP, Perelman JM, Zhou X (2010). Interleukin-13 induces mucin 5AC production involving STAT6/SPDEF in human airway epithelial cells. Cell Commun Adhes.

[62] Vanoni S, Nofziger C, Dossena S, Soyal SM, Patsch W, Plevani P, Duschl A, Paulmichl M (2013). The human pendrin promoter contains two N_4_ GAS motifs with different functional relevance. Cell Physiol Biochem.

[63] Suzuki S, Ogawa M, Ohta S, Nunomura S, Nanri Y, Shiraishi H, Mitamura Y, Yoshihara T, Lee JJ, Izuhara K (2016). Induction of airway allergic inflammation by Hypothiocyanite via epithelial cells. J Biol Chem.

[64] Gernez Y, Tirouvanziam R, Chanez P (2010). Neutrophils in chronic inflammatory airway diseases: can we target them and how?. Eur Respir J.

[65] Kumar RK, Foster PS, Rosenberg HF (2014). Respiratory viral infection, epithelial cytokines, and innate lymphoid cells in asthma exacerbations. J Leukoc Biol.

[66] Aizawa H, Koarai A, Shishikura Y, Yanagisawa S, Yamaya M, Sugiura H, Numakura T, Yamada M, Ichikawa T, Fujino N (2018). Oxidative stress enhances the expression of IL-33 in human airway epithelial cells. Respir Res.

[67] Halwani R, Al-Muhsen S, Al-Jahdali H, Hamid Q (2011). Role of transforming growth factor-beta in airway remodeling in asthma. Am J Respir Cell Mol Biol.

[68] Shifren A, Witt C, Christie C, Castro M (2012). Mechanisms of remodeling in asthmatic airways. J Allergy (Cairo).

[69] Roche WR, Beasley R, Williams JH, Holgate ST (1989). Subepithelial fibrosis in the bronchi of asthmatics. Lancet.

[70] Laitinen A, Altraja A, Kampe M, Linden M, Virtanen I, Laitinen LA (1997). Tenascin is increased in airway basement membrane of asthmatics and decreased by an inhaled steroid. Am J Respir Crit Care Med.

[71] Aubert JD, Dalal BI, Bai TR, Roberts CR, Hayashi S, Hogg JC (1994). Transforming growth factor beta 1 gene expression in human airways. Thorax.

[72] Ohno I, Nitta Y, Yamauchi K, Hoshi H, Honma M, Woolley K, O'Byrne P, Tamura G, Jordana M, Shirato K (1996). Transforming growth factor beta 1 (TGF beta 1) gene expression by eosinophils in asthmatic airway inflammation. Am J Respir Cell Mol Biol.

[73] Vignola AM, Chanez P, Chiappara G, Merendino A, Pace E, Rizzo A, la Rocca AM, Bellia V, Bonsignore G, Bousquet J (1997). Transforming growth factor-beta expression in mucosal biopsies in asthma and chronic bronchitis. Am J Respir Crit Care Med.

[74] Minshall EM, Leung DY, Martin RJ, Song YL, Cameron L, Ernst P, Hamid Q (1997). Eosinophil-associated TGF-beta1 mRNA expression and airways fibrosis in bronchial asthma. Am J Respir Cell Mol Biol.

[75] Yu Q, Stamenkovic I (2000). Cell surface-localized matrix metalloproteinase-9 proteolytically activates TGF-beta and promotes tumor invasion and angiogenesis. Genes Dev.

[76] Robert S, Gicquel T, Victoni T, Valenca S, Barreto E, Bailly-Maitre B, Boichot E, Lagente V (2016). Involvement of matrix metalloproteinases (MMPs) and inflammasome pathway in molecular mechanisms of fibrosis. Biosci Rep.

[77] Su YC, Rolph MS, Hansbro NG, Mackay CR, Sewell WA (2008). Granulocyte-macrophage colony-stimulating factor is required for bronchial eosinophilia in a murine model of allergic airway inflammation. J Immunol.

[78] Uchida K, Beck DC, Yamamoto T, Berclaz PY, Abe S, Staudt MK, Carey BC, Filippi MD, Wert SE, Denson LA (2007). GM-CSF autoantibodies and neutrophil dysfunction in pulmonary alveolar proteinosis. N Engl J Med.

[79] Glasser SW, Detmer EA, Ikegami M, Na CL, Stahlman MT, Whitsett JA (2003). Pneumonitis and emphysema in sp-C gene targeted mice. J Biol Chem.

[80] Gonzalo JA, Lloyd CM, Wen D, Albar JP, Wells TN, Proudfoot A, Martinez AC, Dorf M, Bjerke T, Coyle AJ, Gutierrez-Ramos JC (1998). The coordinated action of CC chemokines in the lung orchestrates allergic inflammation and airway hyperresponsiveness. J Exp Med.

[81] Schneider D, Hong JY, Bowman ER, Chung Y, Nagarkar DR, McHenry CL, Goldsmith AM, Bentley JK, Lewis TC, Hershenson MB (2013). Macrophage/epithelial cell CCL2 contributes to rhinovirus-induced hyperresponsiveness and inflammation in a mouse model of allergic airways disease. Am J Physiol Lung Cell Mol Physiol.

[82] Lukacs NW, Strieter RM, Warmington K, Lincoln P, Chensue SW, Kunkel SL (1997). Differential recruitment of leukocyte populations and alteration of airway hyperreactivity by C-C family chemokines in allergic airway inflammation. J Immunol.

[83] Campbell EM, Charo IF, Kunkel SL, Strieter RM, Boring L, Gosling J, Lukacs NW (1999). Monocyte chemoattractant protein-1 mediates cockroach allergen-induced bronchial hyperreactivity in normal but not CCR2^−/−^ mice: the role of mast cells. J Immunol.

[84] Holla LI, Mrazek F, Petrek M (2009). MCP-1 and CCR2 gene polymorphisms in Czech patients with allergic disorders. Int J Immunogenet.

[85] Srikrishna G (2012). S100A8 and S100A9: new insights into their roles in malignancy. J Innate Immun.

[86] Vogl T, Gharibyan AL, Morozova-Roche LA (2012). Pro-inflammatory S100A8 and S100A9 proteins: self-assembly into multifunctional native and amyloid complexes. Int J Mol Sci.

[87] Kang JH, Hwang SM, Chung IY (2015). S100A8, S100A9 and S100A12 activate airway epithelial cells to produce MUC5AC via extracellular signal-regulated kinase and nuclear factor-kappaB pathways. Immunology.

[88] Chakraborty D, Zenker S, Rossaint J, Holscher A, Pohlen M, Zarbock A, Roth J, Vogl T (2017). Alarmin S100A8 activates alveolar epithelial cells in the context of acute lung injury in a TLR4-dependent manner. Front Immunol.

[89] Hiratsuka S, Ishibashi S, Tomita T, Watanabe A, Akashi-Takamura S, Murakami M, Kijima H, Miyake K, Aburatani H, Maru Y (2013). Primary tumours modulate innate immune signalling to create pre-metastatic vascular hyperpermeability foci. Nat Commun.

[90] Eisenblaetter M, Flores-Borja F, Lee JJ, Wefers C, Smith H, Hueting R, Cooper MS, Blower PJ, Patel D, Rodriguez-Justo M (2017). Visualization of tumor-immune interaction - target-specific imaging of S100A8/A9 reveals pre-metastatic niche establishment. Theranostics.

[91] Rodig T, Endres S, Konietschke F, Zimmermann O, Sydow HG, Wiegand A (2017). Effect of fiber insertion depth on antibacterial efficacy of photodynamic therapy against enterococcus faecalis in rootcanals. Clin Oral Investig.

[92] John AE, Wilson MR, Habgood A, Porte J, Tatler AL, Stavrou A, Miele G, Jolly L, Knox AJ, Takata M (2016). Loss of epithelial G_q_ and G_11_ signaling inhibits TGFbeta production but promotes IL-33-mediated macrophage polarization and emphysema. Sci Signal.

