# Supplementary figures and images for: Loss of the adhesion G-protein coupled receptor ADGRF5 in mice induces airway inflammation and the expression of CCL2 in lung endothelial cells
Source: Respir Res. 2019 Jan 17;20:11. doi: 10.1186/s12931-019-0973-6 (PMC6337809; doi:10.1186/s12931-019-0973-6)

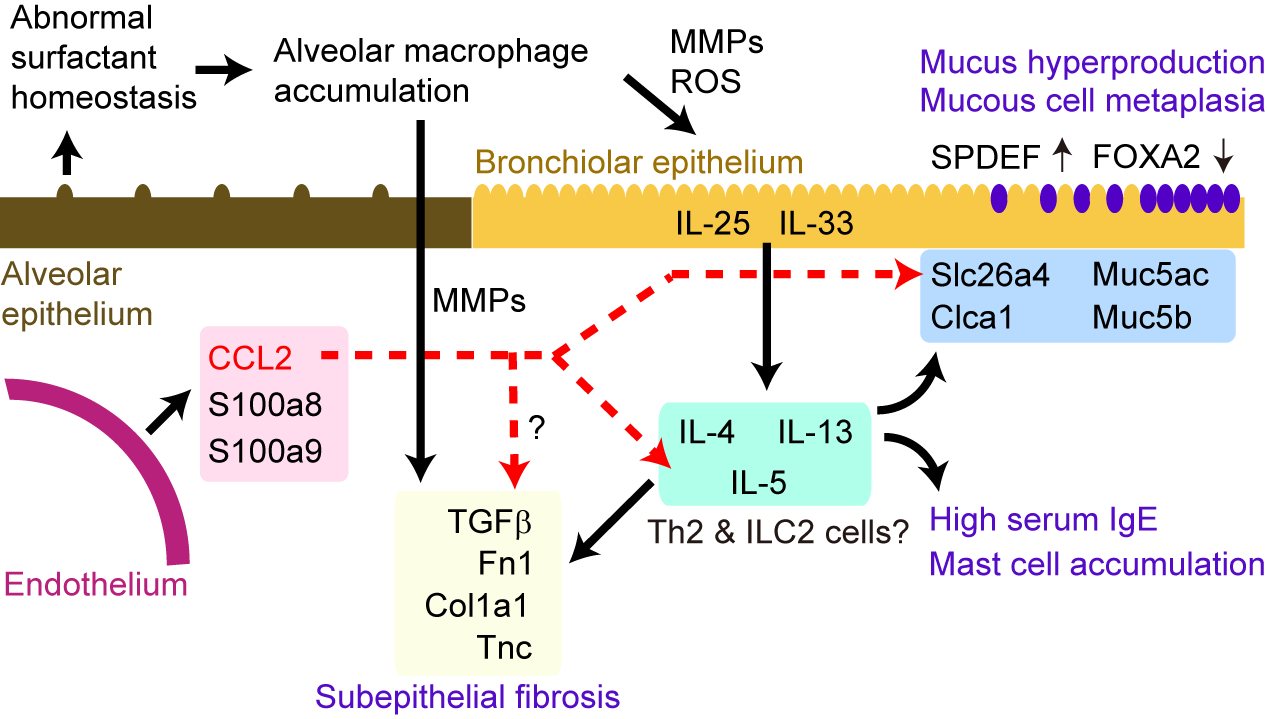

Supplement: Supplementary file 1 — Figure S1. Schematic diagram of a possible mechanism leading to the airway phenotypes of Adgrf5−/− mice. Adgrf5 deletion increases CCL2 expression in the lung endothelium during embryonic and neonatal stages. CCL2 is involved in the upregulation of Slc26a4, Il5, and possibly Tgfb1, thereby contributing to the onset and/or progression of mucus hypersecretion, type 2 inflammation, and fibrosis, respectively (red arrows). Adgrf5 deletion also causes abnormal surfactant homeostasis, which leads to recruitment and activation of alveolar macrophages. MMPs and reactive oxygen species (ROS) released from alveolar macrophages might induce the release of IL-25 and IL-33, and subepithelial fibrosis. IL-25 and IL-33 are likely to increase the production of type 2 cytokines (IL-4, IL-5, and IL-13), which promotes mucous cell metaplasia, mucus hypersecretion, IgE production, mast cell accumulation, and fibrosis. (TIF 208 kb) [file 12931_2019_973_MOESM1_ESM.tif]
